# Exploring the antimicrobial potential of pomegranate peel extracts (PPEs): Extraction techniques and bacterial susceptibility

**DOI:** 10.1371/journal.pone.0315173

**Published:** 2024-12-09

**Authors:** Reem Fawaz Abutayeh, Manal A. K. Ayyash, Ruaa A. Alwany, Alaa Abuodeh, Kamel Jaber, Mohammad A. A. Al-Najjar

**Affiliations:** 1 Faculty of Pharmacy, Department of Pharmaceutical Chemistry and Pharmacognosy, Applied Science Private University, Amman, Jordan; 2 Faculty of Pharmacy, Department of Pharmaceutics and Pharmaceutical Science, Applied Science Private University, Amman, Jordan; 3 School of Medicine, The University of Jordan, Amman, Jordan; Universidad Autonoma de Chihuahua, MEXICO

## Abstract

Antimicrobial resistance is increasing globally and is one of the major public health concerns. This highlights the need to search for new antimicrobial agents. Natural fruit by-products are a rich source of bioactive compounds. Pomegranate (*Punica granatum*) fruit is particularly rich in phenolic bioactive phytochemicals. These compounds are known for their antioxidant, anti-inflammatory, and anticancer properties. Furthermore, they exhibit a broad spectrum of antimicrobial effects. Bioactive phytochemicals are found mainly in peel (exocarp and mesocarp), which constitutes about 50% of the whole fresh fruit. This study utilized pomegranate of Jordanian origin to evaluate the antimicrobial activity of different Pomegranate peel extracts (PPEs) alone and/or in combination with antibacterial agents against four bacterial strains. Different solvents and extraction methods were employed to obtain the PPEs. A key focus was to explore the enhancement of antibacterial activity against gentamicin-resistant *Pseudomonas aeruginosa* (*P*. *aeruginosa*) when microwaved aqueous extracts are combined with gentamicin. The antibacterial activity of PPEs varied depending on the extraction method and the solvent used. Notably, the aqueous macerate and microwave-assisted extract showed high potency and similar activity against *Staphylococcus aureus* (*S*. *aureus*), *Escherichia coli (E*. *coli)*, and *P*. *aeruginosa* (MICs 12.5, 25, and 25 μg/μL, respectively for both aqueous extracts). In contrast, *Proteus mirabilis* (*P*. *mirabilis*) was more susceptible to the inhibitory activity of organic PPEs with a MIC of 25 μg/μL recorded with the use of ethanolic solvents. Bacterial antagonistic activity was observed against gentamicin-resistant *P*. *aeruginosa*, particularly when lower concentrations (3.125, 1.562, 0.781, and 0.39 μg/μL) of microwaved aqueous PPEs were evaluated in combination with different concentrations of gentamicin. In conclusion, pomegranate peels, a natural and safe by-product, demonstrate promising antimicrobial potential. Furthermore, combining PPEs with conventional antibiotics shows promise in addressing antibiotic resistance, highlighting their potential role in treating infectious diseases.

## Introduction

Antimicrobial resistance (AMR) is a critical health threat resulting in hospitalization and death cases annually [1]. AMR is expected to cause 10 million deaths yearly by 2050, making it a major worldwide health problem [2]. Antimicrobial drugs are used to treat a wide range of diseases caused by microorganisms like parasites, fungi, viruses, and bacteria. However, the misuse of these agents, leads to the development of genetic mutations which results in antimicrobial-resistant microorganisms [3]. This resistance results in higher healthcare expenses to cover diagnostic tests, expensive medications and longer treatment durations [4]. The situation is further exacerbated due to the decreasing efficacy of synthetic drugs and their associated toxicity [5].

Scientific attention has turned toward plant-based remedies as potential alternative antimicrobial solutions [6]. Natural products are rich in bioactive secondary metabolites that exhibit promising antimicrobial properties [7]. Recent research has increasingly focused on searching for antimicrobial constituents in plants, fruits, and vegetables. Fruit peels, in particular, are emerging as a prospective source of novel bioactive compounds with antioxidant and antibacterial activity [8].

Pomegranate (*Punica granatum*) is a well-known fruit that is widely consumed. It has been used for thousands of years in traditional medicine to treat various human ailments. It contains a diversity of phyto-active constituents, including alkaloids, flavonoids, tannins, steroids, and triterpenes, which contribute to its reported biological activities [9,10]. In addition to the nutritional value, numerous studies highlight several biological benefits for pomegranate as hypolipidemic, anti-inflammatory, antioxidant, anticancer, antidiabetic, antidiarrheal, anthelminthic, vascular and digestive protection and immunomodulation effects [10,11]. Moreover, pomegranate seeds, peels, and peel extracts have shown significant antimicrobial activity against various bacteria and human pathogens including *Bacillus subtilis*, *E*.*coli*, *Helicobacter pylori*, *Klebsiella pneumoniae*, *Listeria monocytogenes*, *P*. *aeruginosa*, *Salmonella typhi*, *Shigella spp*., *S*.*aureus*, *Candida albicans*, *Clostridium spp*., *Vibrio cholerae*, *Yersinia enterocolitica*, and others microorganisms [12–20].

Recent studies indicate that pomegranate peel extract (PPE) exhibits stronger antimicrobial properties against bacterial and fungal pathogens than other parts of the plant. The antimicrobial activity was related to the total flavonoids and tannins content. While PPE is well known for its antimicrobial activity, it is important to note that the extraction method could significantly influence its efficacy [1].

PPEs, considered as food waste, can be a valuable and sustainable source for the extraction of antimicrobials. A thorough study of the antibacterial properties of PPE can contribute significantly to the development of effective antimicrobial agents as standalone treatments or in combination with conventional antimicrobial drugs to enhance their efficacy [21]. Such an approach offers a countermeasure against the growing challenge of AMR and promotes environmental sustainability by repurposing waste into therapeutic resources.

Microwave-assisted extraction (MAE) is a green technology reported to be effective in recovering bioactive compounds from pomegranate peels [22,23]. Studies have shown that rapid heating and reduced solvent quantities associated with MAE application results in higher yields and better-quality extracts compared to traditional extraction methods [22,24]. Additionally, MAE minimizes the degradation of sensitive phytochemicals such as phenolics, flavonoids and tannins [22,24,25]. While MAE has proved effective as an extraction technique still, limited studies have compared MAE with traditional methods for extracting pomegranate peels and evaluating their antimicrobial efficacy. This gap is particularly important given the potential application of PPEs. Therefore, this work explores the impact of various solvents and extraction techniques, including both conventional and MAE methods, on the antimicrobial activity of PPEs sourced in Jordan.

## Materials and methods

### 1. Plant material

The pomegranate fruit were purchased from a local market in Amman, Jordan. The peels were removed, rinsed, and allowed to shade dry for seven days. The dried peels were ground to fine powder with an electric grinder and stored in an airtight container.

### 2. Preparation of PPE

Dried peels were macerated separately in 1:7.5 and 1:6 weight-to-volume ratios (g/mL) for organic and aqueous solvents, respectively (Table 1). Maceration was applied at room temperature for three days with frequent agitation. Other portions of the dried samples were used to prepare aqueous decoctions and an infusion of PPE in 1:8 weight-to-volume ratio (g/mL). Aqueous decoctions were prepared by two separate methods; one was prepared with the use of a microwave (Sharp^®^, Model: R-340D, Serial number:010703004) for 8 min with power set at 900 watts (microwave assisted extract, MAE) and the other was prepared by regular boiling of the dried peels in water for 20 min. The aqueous infusion was prepared by adding the dried peel to boiled water and covering it for 10 min. All extracts were filtered using Whatman No.1 filter paper. Organic PPEs (i.e., absolute ethanol, 50% ethanol, and acetone extracts) were concentrated under reduced pressure using a rotary evaporator at 40°C until complete evaporation. Aqueous PPEs including the macerate, decoction and MAE were concentrated using a freeze drier. The crude PPEs were kept in the dark at 4°C until needed.

**Table 1:** Extraction yield of pomegranate extracts.

Solvent	Wt. of extract (gm)	% Yield
50% ethanol	16	8
Absolute ethanol	9.7	4.9
Acetone	9.2	4.6
Water (maceration)	2.75	5.5
Water (decoction)	0.85	6.8
Water (infusion)	1	8
Water (microwave-assisted)	1.25	10

The extraction yield was calculated using the following formula:

Extractionyield=massofextract(gm)*100/massofdrymatter(gm)

The extraction yield ranged from 4.6% for the acetone PPE up to 10% for the MA-PPE.

### 3. Microdilution Minimum Inhibition Concentration (MIC) determination

Seven extracts that include organic and aqueous PPEs were screened against four strains of bacteria: three-Gram negative bacteria (*E*. *coli* "ATCC 14169", *P*. *aeruginosa* "ATCC 27853" and *P*. *mirabilis* "ATCC 29245"), and one Gram-positive bacteria (*S*. *aureus* "ATCC 25923"), were procured from the American Type Culture Collection (ATCC, USA) as shown in Tables 3 and 4.

Each bacterial stock suspension was prepared by inoculating the bacterial strains in nutrient broth. The suspensions were incubated at 37°C overnight and were then standardized to 10^8^ colony forming units per milliliter (CFU/mL) equivalent to the turbidity of 0.5 McFarland standard for each bacterium, respectively.

The MIC of all organic and aqueous PPEs was determined using the broth microdilution method in 96-well microplates. Each PPE was diluted in sterilized Nutrient broth (NB) to the highest concentration (50μg/μL) to be tested, and then serial two-fold dilutions were made. A series of eight dilutions of the individual PPEs were prepared ranging from a concentration of 0.39μg//μL to 50μg//μL. The microplate was prepared by dispensing 100/μL of the PPE solutions respectively and 100/μL of standardized bacterial suspension. The negative control wells contained only the tested bacteria in NB. Blank wells contained the various dilutions of each respective PPE without any bacteria. The final volume in all wells was adjusted to 200 /μL, when needed. The microplates were then incubated for 24h at 37°C, and absorbance was measured at 600nm using an ELISA microplate reader. All these analyses were performed in triplicates. The MIC was defined as the lowest concentration of a particular PPE to inhibit the growth of microorganisms. The antibacterial efficacy of each extract was quantified by measuring the concentration of viable bacterial cells, expressed as CFU/mL, across the range of extract dilutions. The extent of bacterial growth inhibition was determined by calculating the logarithmic reduction in CFU/mL, relative to the initial bacterial concentration.

#### Antibiotic susceptibility test

Susceptibility testing was performed for the four bacterial strains against three antibiotics: Cefixime, Ciprofloxacin, and Gentamicin. The MIC for each antibiotic was assessed using a broth microdilution method in 96-well microplates. This involved preparing serial dilutions of each antibiotic and adding them to wells containing standardized bacterial suspensions. The assay setup, incubation conditions, and measurements were akin to those described for the PPEs, with MIC values determined based on the lowest concentration of antibiotic that prevented bacterial growth.

### 4. Evaluation of the inhibitory activities of aqueous MA-PPE-Gentamicin combinations against resistant *P*. *aeruginosa*

The potential of enhanced antibacterial activity of MA-PPE in conjunction with Gentamicin against *P*. *aeruginosa* was assessed utilizing the checkerboard technique. This involved preparing eight distinct dilutions of the aqueous MA-PPE, ranging from 0.39 to 50 μg//μL combined with a range of equivalent concentrations of Gentamicin, resulting in a total of 64 unique combinations. Each combination was methodically tested against *P*. *aeruginosa* to evaluate the potential enhanced effects and establish an effective antimicrobial strategy.

### 5. Data analysis

Statistical analyses were performed using SPSS version 26 (IBM Corp. Armonk, NY, USA). All analyses were carried out in triplicates and the results were presented as means ± standard deviations. Non-parametric statistical tools were used, as the data did not follow a normal distribution confirmed by Shapiro-Wilk test (p<0.05) (S2 File). The Mann-Whitney U test was used to compare CFU/mL between organic-PPEs versus aqueous-PPEs across different concentration levels. The Kruskal-Wallis One-way ANOVA test was used to compare the effect of the seven different solvents, the four bacterial strains and the different concentrations (using three concentration levels: 50, 25, and 12.5 μg/μL) on CFU/mL. Post-hoc pairwise comparisons were conducted using Dunn’s test, followed by Bonferroni’s correction for multiple tests. Differences between tested groups were considered significant at *P <* 0.05. Detailed statistical analyses are provided in the (S3 File).

## Results

### PPE extraction yield

The extraction yield varied according to the solvent and method used. Comparing the yields achieved through the maceration technique, the 50% ethanol macerate exhibited the highest yield of 8% followed by water (5.5%), absolute ethanol (4.9%), and lastly acetone macerate with the lowest yield of 4.6%. Furthermore, extraction yields notably differed among the aqueous PPEs, depending on method employed. The highest yield was achieved with the utilization of the MAE method (10%) followed by infusion (8%), decoction (6.8%), and maceration (5.5%). These results demonstrate a range in extraction efficiency from a low yield of 4.6% for acetone macerate to a high yield of 10% for the MA-PPE, as shown in Table 1.

### Minimum inhibitory concentration (MIC)

The MIC of all organic and aqueous PPEs was determined using the broth microdilution method in 96-well microplates as explained in Methodology section. A series of eight dilutions of the individual PPEs were prepared ranging from a concentration of 0.39μg/μL to 50μg/μL. The microplate was prepared by dispensing 100/μL of the PPE solutions respectively and 100/μL of standardized bacterial suspension. The negative control wells contained only the tested bacteria in NB. Blank wells contained the various dilutions of each respective PPE without any bacteria. The microplates were then incubated for 24h at 37°C, and absorbance was measured at 600nm using an ELISA microplate reader. All these analyses were performed in triplicates (S1 File).

### MICs of antibiotics

MIC of three antibiotics was assessed against four types of microorganisms. The analyses were performed in triplicates and the result is shown in Table 2.

**Table 2:** MIC of the antibiotics against four types of bacterial strains.

**Bacterial strain**		MIC (μg/μL)		
		**Ciprofloxacin**	**Cefixime**	**Gentamicin**
	*S*. *aureus*	≤0.39	3.125	> 0.39
	*E*. *coli*	25	≤0.39	> 0.39
	*P*. *aeruginosa*	25	6.25	Resistant
	*P*. *mirabilis*	12.5	≤0.39	Resistant

### MIC of PPEs

This study assessed the antibacterial activities of seven distinct PPEs that were evaluated against four bacterial strains, with the results detailed in Tables 3 and 4. It is noted that the solvent polarity, extraction method, as well as the susceptibility of the bacterial strains are all crucial factors in MIC results. The organic- PPEs (50% ethanol, absolute ethanol and acetone) represent different polarities under the same method of extraction. While the aqueous-PPEs (macerate, MA-PPE, infusion and decoction) represent different extraction methods with constant polarity in all four PPEs.

**Table 3:** MIC of organic-PPEs against four types of bacteria.

Solvent	MIC (μg/μL)			
	*S*. *aureus*	*E*. *coli*	*P*. *aeruginosa*	*P*. *mirabilis*
**50% ethanol**	25	50	50	25
**Absolute ethanol**	Inactive	Inactive	50	25
**Acetone**	25	50	50	50

**Table 4:** MIC of aqueous-PPEs against four types of bacteria.

Solvent	MIC (μg/μL)			
	*S*. *aureus*	*E*. *coli*	*P*. *aeruginosa*	*P*. *mirabilis*
**Maceration**	12.5	25	25	Inactive
**Decoction**	Inactive	50	Inactive	Inactive
**Infusion**	50	50	50	Inactive
**Microwave- assisted (MAE)**	12.5	25	25	50

The organic PPEs demonstrated varying levels of MIC, and the 50% ethanol PPE inhibited the growth of all four bacterial strains and was the highest performing solvent among the organic- PPEs. Similarly, the MA-PPE was the most efficacious among the aqueous–PPEs particularly against *S*. *aureus*, and *P*. *aeruginosa* (MICs of 12.5 and 25 μg/μL respectively). However, it was less potent against *P*. *mirabilis* compared to the 50% ethanol extract. Noteworthy, when comparing the CFU/mL readings in both 50% ethanol and MA-PPE at three concentration levels (50, 25, and 12.5 μg/μL), there is no statistical difference between the two extracts in all four strains of bacteria (S3 File). *P*. *aeruginosa* showed similar sensitivity to different organic-PPEs regardless of the solvents’ polarities, but it was more sensitive to the extraction techniques (using the same polarity) where it was more susceptible to the macerate and MA-PPE (MIC 25 μg/μL for both techniques) rather than the infusion (MIC 50 μg/μL) or decoction (inactive) techniques. On the other hand, *P*. *mirabilis* is more sensitive to the polarity of the solvents rather than the techniques. It seems to be responding to the PPEs within a certain range of polarity, losing its sensitivity to the highly polar aqueous macerate and to the least polar organic–PPE (i.e. acetone). Notably, the macerate and the MA-PPE have equal MICs on all strains respectively, except for *P*. *mirabilis*, where the MAE was still efficacious at MIC of 50 μg/μL while the macerate was inactive. *E*. *coli* and *S*. *aureus* are affected by both the solvent polarity and the extraction technique.

**Fig 1:**
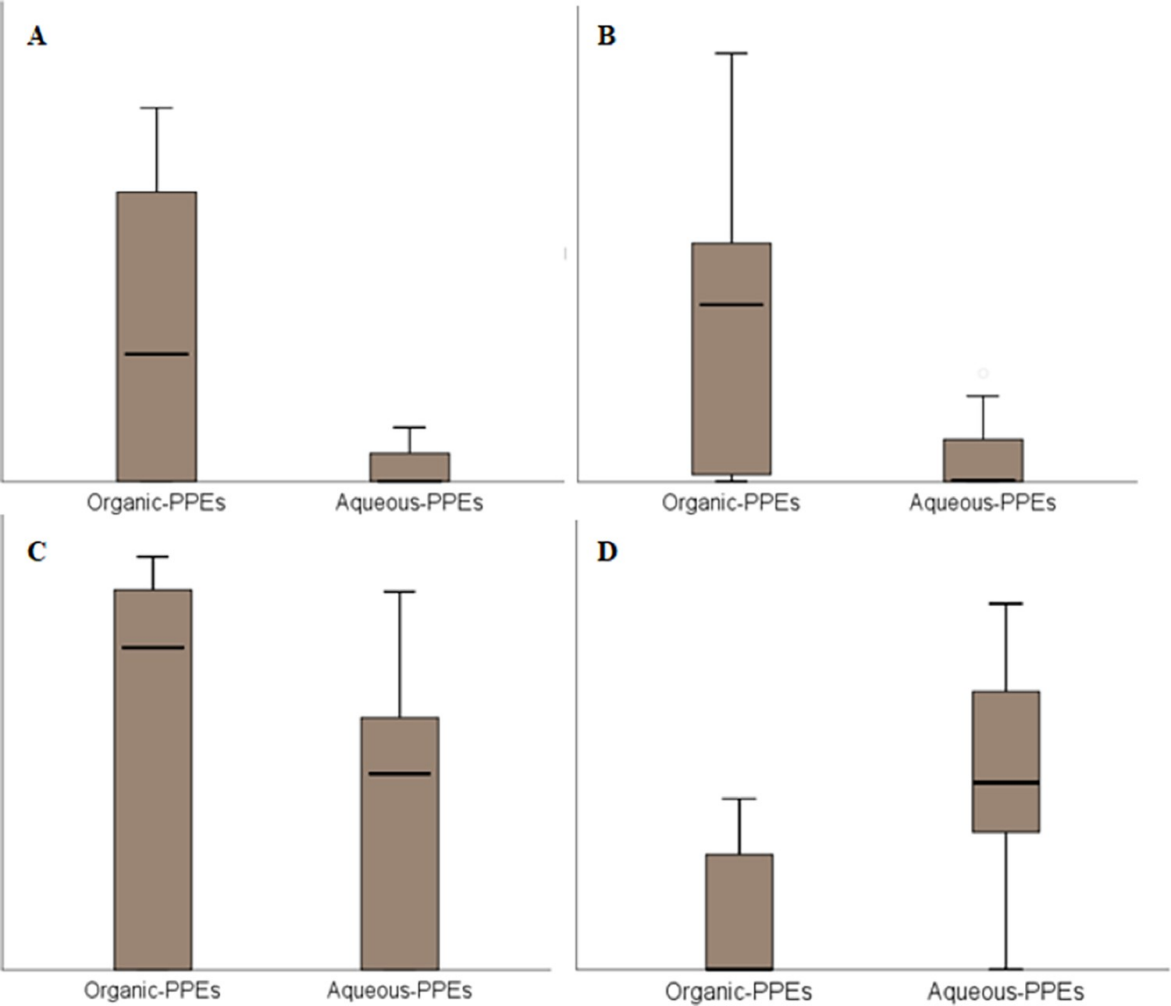
Distribution of CFU/mL across extract types (organic versus aqueous) in A: *S*. *aureus*, B: *E*. *coli*, C: *P*. *aeruginosa* and D: *P*. *mirabilis*.

Data analysis of triplicate CFU/mL values was performed for each bacterial strain to check for differences in antimicrobial efficacy across the seven solvents at the highest three levels of concentration **(**50, 25, and 12.5 μg/μL), using non-parametric statistical tools. The results illustrated significant differences in CFU/mL according to extract types (organic-PPEs versus aqueous-PPEs). Where aqueous -PPEs were significantly more effective in reducing the levels of CFU/mL in *S*. *aureus* [z = -2.252, p = .024], *E*. *coli* [z = -4.101, p < .001], and *P*. *aeruginosa* [z = -2.43, p = .015]. While organic-PPEs were significantly more effective in in reducing the levels of CFU/mL in *P*. *mirabilis* [z = -4.771, p< .001], as shown in Fig 1.

### Antibacterial activity of PPEs against *S*. *aureus*

**Fig 2:**
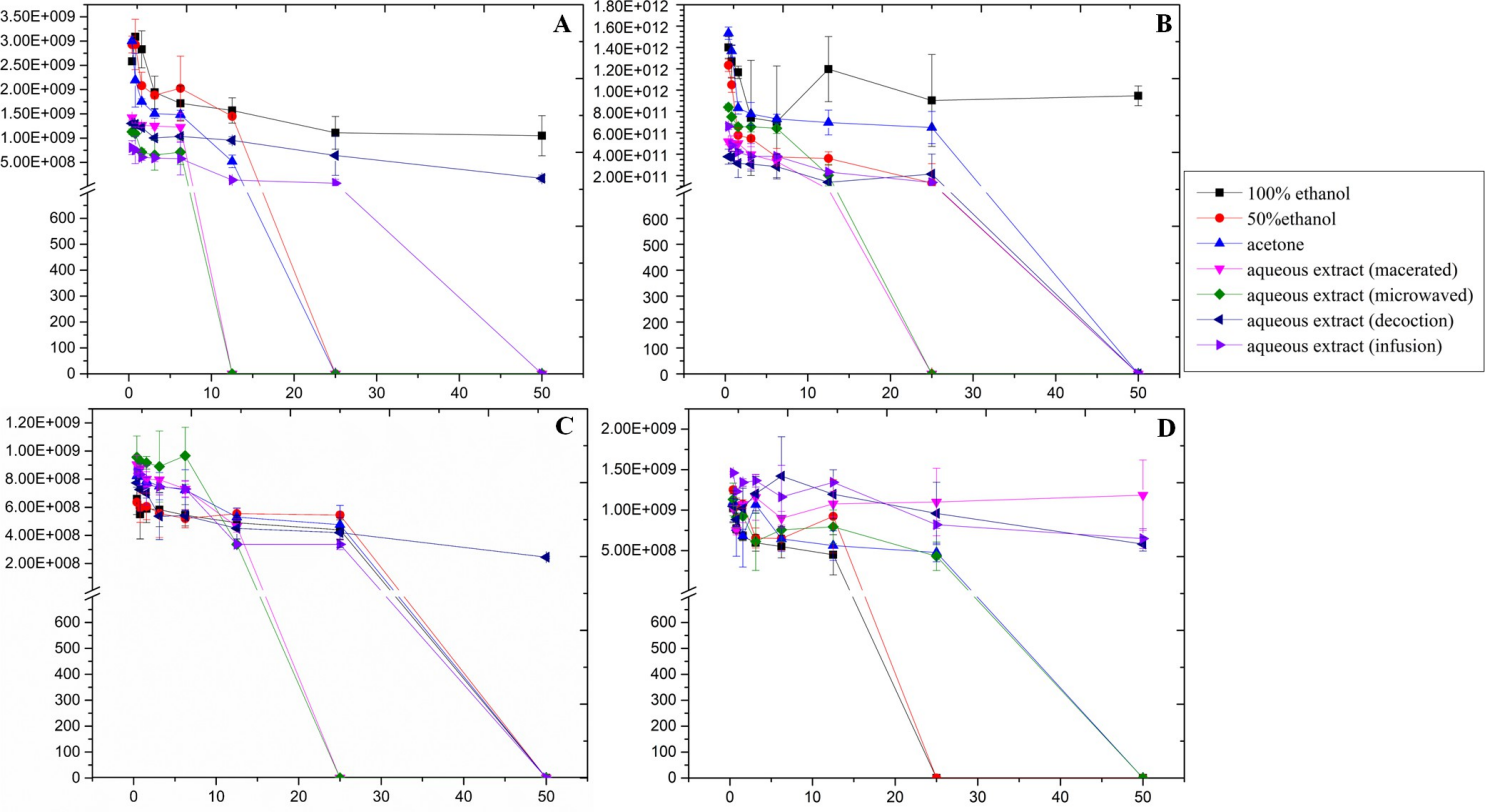
Comparison of the antibacterial activities of seven PPEs against A: *S*. *aureus*. B: *E*. *coli*, *C*: *P*. *aeruginosa*, *D*: *P*. *mirabilis*.

The evaluation of PPE activities against *S*. *aureus* revealed a range of antibacterial activities, depending on extraction method and /or solvent used as shown in Fig 2A. The aqueous PPEs, obtained through maceration and MAE techniques, showed the highest potency of MIC of 12.5 μg/μL. The organic PPEs, particularly those using 50% ethanol and acetone solvents, also demonstrated considerable antibacterial properties, inhibiting *S*. *aureus* growth at a MIC of 25 μg/μL. The aqueous PPE produced by infusion exhibited a higher MIC of 50 μg/μL, suggesting reduced effectiveness against this bacterium. In addition, the absolute ethanol PPE and the aqueous decoction were found to be inactive, indicating their limited efficacy against *S*. *aureus*.

**Fig 3:**
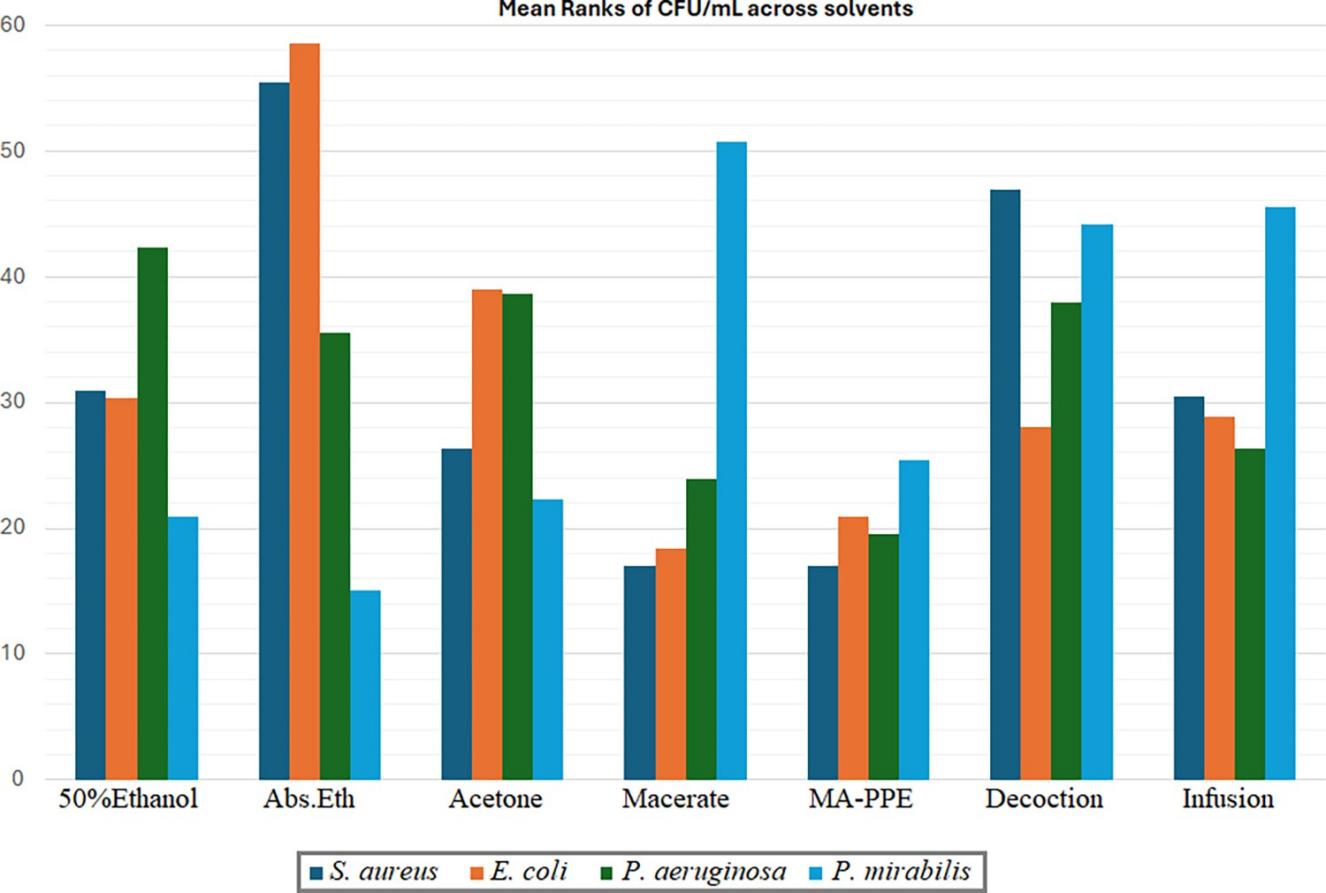
Mean ranks comparison of CFU/mL in different bacterial strains distributed across seven types of solvents.

Statistical analysis reinforced these observations, showing significant differences in antimicrobial efficacy across solvents across different solvents (shown in Fig 3). The macerate and MA-PPE aqueous extracts displayed the lowest CFU/mL mean ranks (both at 17), indicating greater effectiveness compared to other solvents. The mean rank for both solvents was significantly lower than absolute ethanol (mean rank 55.44) with p–values < 0.001 for macerate and MA-PPE. Furthermore, absolute ethanol results were significantly higher than that of the infusion (p = 0.04), 50% ethanol (p = 0.045), and acetone (p = 0.006). As for the decoction, the CFU/mL mean rank was also significantly higher than those of macerate and MA-PPE (p = 0.004 for both comparisons).

### Antibacterial activity of PPEs against *E*. *coli*

Except for the absolute ethanol extract, all PPEs demonstrated inhibitory effects on *E*. *coli* growth, albeit with varying MICs. Notably, the aqueous PPE macerate, the MA-PPE and the 50% ethanol extract were particularly effective, inhibiting bacterial growth at a concentration of 25μg/μL. While the remaining three active PPEs showed inhibitory activity at a higher concentration of 50μg/μL, as illustrated in Fig 2B.

The statistical analysis verified these observations, showing that absolute ethanol had a significantly higher CFU/mL mean rank (58.5) than all aqueous PPEs (macerate, MA-PPE, decoction, and infusion), with p-values ranging from <0.001 to 0.009, respectively. In addition, absolute ethanol differed significantly than the 50% ethanol extract (p = 0.017) as can be seen in Fig 3. This further highlights the enhanced efficacy of aqueous extracts for *E*. *coli* compared to organic PPEs, where the macerate and MA-PPE extracts exhibited the lowest mean ranks (18.4 and 20.9, respectively), indicating higher antibacterial efficacy against *E*. *coli*.

### Antibacterial activity of PPEs against *P*. *aeruginosa*

The antibacterial activity of different PPEs against *P*. *aeruginosa* exhibited a range of inhibitory effects, as shown in Fig 2C. Like the sensitivity pattern observed in *S*. *aureus*, the *P*. *aeruginosa* growth was more sensitive to the macerate and the MA-PPE, each demonstrating an MIC of 25 μg/μL, a finding comparable to the MICs reported for *E*. *coli*. The other four active extracts, which included the organic and infusion-based aqueous PPEs, required higher concentrations (MIC of 50 μg/μL) to inhibit the growth of *P*. *aeruginosa*. Notably, the PPE prepared by the decoction method was inactive against *P*. *aeruginosa* with results similar to that of *S*. *aureus*. Interestingly, the absolute ethanol extract, inactive against *S*. *aureus* and *E*. *coli*, displayed antibacterial activity against *P*. *aeruginosa* with an MIC of 50 μg/μL.

In the case of *P*. *aeruginosa*, no significant differences were observed for the CFU/mL distribution across the individual solvents (Fig 3) when applying Bonferroni correction for multiple tests. This does not align with earlier results based on extract type comparisons. This means that further investigations are needed such as including additional concentration levels in the comparisons. Still, the aqueous extracts including MA-PPE, macerate, and infusion exhibited the lowest CFU/mL mean ranks (19.6, 23.9, and 26.3 respectively), yet these differences were not statistically significant.

### Antibacterial activity of PPEs against *P*. *mirabilis*

In the assessment of PPEs against *P*. *mirabilis*, four extracts were found to be effective (Fig 2D). These included the three organic extracts (100% ethanol, 50% ethanol, and acetone) and the aqueous MA-PPE. The microwave-assisted and acetone extracts displayed antibacterial activity at a concentration of 50 μg/μL. In contrast, both the 50% and 100% ethanol extracts exhibited a higher potency against this bacterium, inhibiting its growth at a lower concentration of 25 μg/μL.

Statistical analysis highlighted strong differences between organic and aqueous PPEs (Fig 3), with organic PPEs generally being more effective against *P*. *mirabilis*. Notably, the lowest CFU/mL mean rank was observed in absolute ethanol (15.11), followed by MA-PPE (25.39). This indicates that while *P*. *mirabilis* showed a preference for organic PPEs, MA-PPE was still highly effective, with no significant difference in CFU/mL levels compared to absolute ethanol. Additional post-hoc comparisons indicated significant differences between pairs such as absolute ethanol-decoction (p = 0.014), absolute ethanol-infusion (p = 0.008), and absolute ethanol-macerate (p = 0.001).

### Evaluation of the inhibitory activities of aqueous MA-PPE-Gentamicin combinations against resistant *P*. *aeruginosa*

In this part of our study, the potential enhanced antibacterial activity of MA-PPE in combination with gentamicin was investigated against gentamicin-resistant *P*. *aeruginosa* strain. The decision to use MA-PPE was based on its notable efficacy in inhibiting *P*. *aeruginosa* at a relatively low MIC of 25 μg/μL. A comprehensive array of 64 combinations was created by mixing eight dilutions of aqueous MA-PPEs (50, 25, 12.5, 6.25, 3.125, 1.562, 0.781, and 0.39 μg/μL) with the same dilutions of gentamicin. These combinations were tested against *P*. *aeruginosa* one at a time, and each test was repeated three times. A bactericidal effect was observed when gentamicin was paired with the four lower concentrations of MA-PPE (3.125, 1.562, 0.781, and 0.39 μg/μL). In contrast, combinations where gentamicin was mixed with the higher concentrations of MA-PPE (50, 25, 12.5, 6.25 μg/μL), did not exhibit antibacterial activity.

## Discussion

Natural sources, particularly plants and their bioactive constituents, are being explored as alternatives or supplements to conventional antimicrobial agents due to the urgent need for effective treatments for bacterial infections and to address the growing issue of antimicrobial resistance (AMR). In the search for practical alternatives many studies have evaluated the antimicrobial effects of natural sources, either alone or in combinations such as pomegranate [1,9–20], cranberry [26], guava [27], and sage [27,28].

Pomegranate including its peels, seeds and fruit juice, are promising sources of bioactive metabolites, with notable antioxidants and antimicrobial activities [29]. Our findings on the antimicrobial activity of PPEs against a variety of bacteria, including antibiotic-resistant strains, aligns with the extensive study done by Kupnik et al [29] that proved the significant efficacy of *P*. *granatum* in inhibiting bacterial growth across multiple bacterial strains. Our study, focused on peel extracts, underscores the significance of PPEs in combating bacterial resistance and reinforces their potential as a source of natural antimicrobial agents, particularly when using MAE methods. The potential for pomegranate (peels, seeds and juice) to contribute to broader therapeutic applications still is an exciting avenue for future exploration.

Punicalagin, a polyphenol, was mainly accredited for the antimicrobial activity of pomegranate peel extracts [30,31]. The qualitative phytochemical screening of local pomegranate peels done by Sweidan et al [20] showed the presence of phenols, flavonoids, anthocyanins, coumarins, quinones, tannins, saponins, steroids, triterpenoid, and alkaloids, as general phytochemical classes. This suggests a need for qualitative and quantitative analyses of individual PPE constituents, such as punicalagins, in local Jordanian pomegranate peels to better understand their antimicrobial and antioxidant mechanisms.

The diverse bioactive compounds in plants can be extracted using various common methods such as maceration, decoction, infusion, and MAE [32]. Optimizing the extraction conditions -including solvent polarity, solvent-to-solid ratios, extraction temperatures, and extraction duration- can enhance the efficiency and yield of phenolic compounds from pomegranate peels. Consistent with earlier research that compared various solvents in the extraction of pomegranate peels [30,31,33], our study reaffirms the importance of solvent selection in maximizing bioactive compound yield. While methanol is often cited as an effective solvent for phenolic extraction, water also shows large efficacy, offering a more environmentally friendly, sustainable and cost-effective alternative [33]. Consistent with previous studies [23,24], our findings demonstrate the advantages of MAE where MAE-PPE’s yield was the highest, followed by the aqueous infusion and decoction.

Although higher yield does not necessarily correlate with better activity (as seen in the decoction extract), the MAE-PPE’s antimicrobial activity was compared with other conventional extracts including organic and aqueous PPEs and it exhibited a consistent efficacy across all tested bacterial strains, which proves that this method preserves the constituents’ integrity needed for antimicrobial activity. In general, aqueous-PPEs showed better performance than the organic-PPEs, across most bacterial strains, except for *P*. *mirabilis*, where the aqueous PPEs were inactive except for MA-PPE (MIC 50 μg/μL), which maintained a relatively strong antimicrobial activity and was not significantly different from the highly effective 100% ethanol extract (MIC 25 μg/μL). This suggests that MA-PPE may possess certain bioactive properties that enable it to retain efficacy against *P*. *mirabilis* at a level similar to that of 100% ethanol.

In our assessment of the antibacterial efficacy of PPEs compared to conventional antibiotics, the MIC of ciprofloxacin (MIC 25 μg/μL) was found to be equal to that of the aqueous macerate and the MA-PPE against both *P*. *aeruginosa* and *E*. *coli* and equal to the 50% ethanol extract against *E*. *coli*. Cefixime showed superior activity to PPEs, while gentamicin was inactive against *P*. *mirabilis* and *P*. *aeruginosa*.

*P*. *aeruginosa* is a Gram- negative bacterium with high resistance patterns to antibiotics. It is often reported in cases of community-acquired pneumonia, specifically in hospitalized patients or those in nursing homes, and is associated with severe illnesses and poor clinical outcomes [34]. It is also listed in the WHO (World Health Organization) priority group 1 of pathogens that urgently require new treatment options [35]. Contrary to gentamicin, six PPEs were active against *P*. *aeruginosa* at MICs of 25 and 50 μg/μL for the organic and aqueous PPEs, respectively (Table 3). Furthermore, using low concentrations of MA-PPE (range 0.39–3.125 μg/μL) in combination with gentamicin showed bactericidal activity in gentamicin-resistant *P aeruginosa*. Resistance to gentamicin can be caused by several factors including: 1) enzymatic inactivation, 2) reduced uptake of gentamicin due to changes in outer membrane porins or overexpression of efflux pumps, 4) ribosomal mutation and/or 5) biofilm formation [36]. The increased sensitivity to gentamicin when combined with MA-PPE can be related to any one of these resistance mechanisms, where low doses of MA-PPE may enhance the permeability of the bacterial cell membrane or inhibit efflux pumps, facilitating the uptake of gentamicin more effectively [37]. At higher concentration of MA-PPE, an antagonistic activity is observed, where neither the MA-PPE nor gentamicin was active. MA-PPE, being a complex mixture containing multiple compounds, potentially has multiple mechanisms of action [37]. Some of these mechanisms may be antagonized by gentamicin or other constituents within the extract. Antagonistic activity might also be due to saturation of the biological targets in the presence of gentamicin and MA-PPE in high concentration. The loss of efficacy might be due to several factors and this point needs to be investigated further. Although the antimicrobial activity of PPEs in combination with gentamicin has not been widely explored, it is worth noting that pomegranate was found to be protective against gentamicin-induced nephrotoxicity [38] and ototoxicity [39] in animal models. Using PPEs to increase sensitivity of *P*. *aeruginosa* to gentamicin is a promising application in hospitals either as adjuvant therapy or even as a disinfectant.

A recent study addressed the antimicrobial activity of local Jordanian PPEs against seven microbial strains including five bacterial and two fungal strains. The PPEs were prepared by maceration using six solvents with different polarities (i.e., water, methanol, aqueous methanol, ethanol, ethyl acetate, and butanol). The best antimicrobial performance was achieved by the ethanol and methanol extracts followed by the aqueous methanol and water extracts, while the least polar solvents lacked any antimicrobial activity [20]. Although our study differs in the bacterial strains covered and the range of polarities of extracting solvents, both studies have worked on *P*. *aeruginosa* and include ethanol and water extracts of pomegranate peels. Their findings concerning the antimicrobial activity of PPEs are supported by our results, where both ethanol (50% and 100% ethanol) extracts were active against *P*. *aeruginosa*. However, our findings differ in that the aqueous macerate and the MA-PPE performed better than the ethanol extracts. Moreover, our study went a step further in exploring the effect of PPEs when combined with gentamicin on *P*. *aeruginosa*.

Both studies show the promising antimicrobial activity of Jordanian PPEs. Differences in results could be attributed to the pomegranate cultivar, geographical origin, and extraction methods, all of which can influence the phytochemical composition and concentrations in the extracts.

## Conclusion

This study is the first study to address the antimicrobial activity of MAE of Jordanian pomegranate peels and compare it to traditional methods of extraction. The results affirm MAE as an efficient alternative with improved recovery of bioactive constituents.

The study also highlighted the potential antimicrobial activity of different PPEs by evaluating their MICs against four bacterial strains including gentamicin-resistant *P*. *aeruginosa*. The MA-PPE displayed remarkable results, showing potential as an adjuvant treatment, as it enhanced the efficacy of gentamicin. The findings underline the application of antimicrobial PPEs as standalone agents or in combination with other antimicrobial agents like gentamicin. The enhanced activity of the PPE-gentamicin combination against gentamicin-resistant *P*. *aeruginosa* underscores the importance of natural products in addressing the global challenge of AMR. Notably, our research supports green, eco-friendly practices by using food waste like pomegranate peels as a source for antimicrobial agents.

Future research should focus on identifying the bioactive constituents of Jordanian PPEs and exploring their mechanisms of action. In addition, extraction methods need to be optimized to grasp the full antimicrobial potential of PPEs. To validate the applicability of PPEs as antimicrobial treatment or co-treatment, upcoming research must broaden the spectrum of tested bacterial strains and other pathogens and must assess the efficacy of PPEs in preclinical and clinical settings.

## Supporting information

S1 File Antimicrobial activity data of PPEs against *S*. *aureus*, *E*. *coli*, *P*. *aeruginosa*, and *P*. *mirabilis*. (PDF)

S2 File Statistical analysis -normality of input data. (PDF)

S3 File Statistical analysis- non-parametric analyses of data. (PDF)
