# Supplementary material for: Exploring the antimicrobial potential of pomegranate peel extracts (PPEs): Extraction techniques and bacterial susceptibility
Source: PLoS One. 2024 Dec 9;19(12):e0315173. doi: 10.1371/journal.pone.0315173 (PMC11627421; doi:10.1371/journal.pone.0315173)
Supplement: S1 File — (PDF) [file pone.0315173.s001.pdf]

Antimicrobial activity against S.aureus										
	100% ethanol					50%ethanol				
conc.	trial 1	trial 2	trial 3	average	STD	trial 1	trial 2	trial 3	average	STD
50	9.9E+08	6.7E+08	1.5E+09	1.05E+09	4.1E+08	0	0	0	0	0
25	8.5E+08	9.9E+08	1.5E+09	1.11E+09	3.4E+08	0	0	0	0	0
12.5	1.27E+09	1.7E+09	1.7E+09	1.57E+09	2.6E+08	1.41E+09	1.51E+09	1.43E+09	1.45E+09	52915026
6.25	1.45E+09	1.6E+09	2.1E+09	1.72E+09	3.6E+08	2.43E+09	2.39E+09	1.26E+09	2.03E+09	6.64E+08
3.125	2.33E+09	1.8E+09	1.8E+09	1.94E+09	3.3E+08	1.93E+09	1.93E+09	1.8E+09	1.89E+09	75055535
1.562	2.39E+09	3.1E+09	3.1E+09	2.83E+09	3.8E+08	2.13E+09	2.33E+09	1.79E+09	2.08E+09	2.73E+08
0.781	3.13E+09	3.1E+09	3.1E+09	3.09E+09	3.5E+07	2.33E+09	3.23E+09	3.23E+09	2.93E+09	5.2E+08
0.39	2.65E+09	2.6E+09	2.5E+09	2.58E+09	6.1E+07	2.83E+09	2.83E+09	3.13E+09	2.93E+09	1.73E+08
	acetone					aqueous extract (macerated)				
conc.	trial 1	trial 2	trial 3	average	STD	trial 1	trial 2	trial 3	average	STD
50	0	0	0	0	0	0	0	0	0	0
25	0	0	0	0	0	0	0	0	0	0
12.5	3.7E+08	6E+08	5.9E+08	5.2E+08	1.3E+08	0	0	0	0	0
6.25	1.39E+09	1.5E+09	1.6E+09	1.48E+09	9E+07	8.9E+08	1.37E+09	1.41E+09	1.22E+09	2.89E+08
3.125	1.61E+09	1.5E+09	1.5E+09	1.5E+09	9.2E+07	1.19E+09	1.27E+09	1.29E+09	1.25E+09	52915026
1.562	1.78E+09	1.8E+09	1.7E+09	1.75E+09	5.5E+07	1.25E+09	1.23E+09	1.31E+09	1.26E+09	41633320
0.781	1.82E+09	2.8E+09	1.9E+09	2.19E+09	5.5E+08	1.27E+09	1.31E+09	1.23E+09	1.27E+09	40000000
0.39	2.97E+09	3E+09	3E+09	3E+09	3.1E+07	1.37E+09	1.45E+09	1.45E+09	1.42E+09	46188022
	aqueous extract (microwaved)					aqueous extract (decoction)				
conc.	trial 1	trial 2	trial 3	average	STD	trial 1	trial 2	trial 3	average	STD
50	0	0	0	0	0	2.5E+08	1.3E+08	1.3E+08	1.7E+08	69282032
25	0	0	0	0	0	2.1E+08	1.03E+09	6.9E+08	6.43E+08	4.12E+08
12.5	0	0	0	0	0	8.7E+08	1E+09	9.94E+08	9.55E+08	73384830
6.25	9.9E+08	6.5E+08	4.9E+08	7.1E+08	2.6E+08	1.05E+09	1.07E+09	9.9E+08	1.04E+09	41633320
3.125	6.7E+08	3.3E+08	9.5E+08	6.5E+08	3.1E+08	1.05E+09	1.01E+09	9.5E+08	1E+09	50332230
1.562	6.3E+08	7.5E+08	7.5E+08	7.1E+08	6.9E+07	1.25E+09	1.21E+09	1.19E+09	1.22E+09	30550505
0.781	1.15E+09	1.1E+09	1.1E+09	1.09E+09	5.1E+07	1.21E+09	1.37E+09	1.27E+09	1.28E+09	80829038
0.39	1.07E+09	1.2E+09	1.1E+09	1.13E+09	6E+07	1.35E+09	1.25E+09	1.31E+09	1.3E+09	50332230

aqueous extract (infusion)					
**conc.**	**trial 1**	**trial 2**	**trial 3**	**average**	**STD**
50	0	0	0	0	0
25	12500000	1.3E+08		68750000	8E+07
12.5	1.5E+08	1.3E+08	1.3E+08	1.37E+08	1.2E+07
6.25	7.9E+08	1.9E+08	7.5E+08	5.77E+08	3.4E+08
3.125	5.1E+08	6.5E+08	6.1E+08	5.9E+08	7.2E+07
1.562	5.5E+08	5.9E+08	6.7E+08	6.03E+08	6.1E+07
0.781	4.7E+08	1E+09	7.7E+08	7.57E+08	2.8E+08
0.39	7.1E+08	9.7E+08	7.3E+08	8.03E+08	1.4E+08

Antimicrobial activity against e.coli										
	100% ethanol					50%ethanol				
conc.	trial 1	trial 2	trial 3	average	STD	trial 1	trial 2	trial 3	average	STD
50	1E+12	8.4E+11	1E+12	9.4667E+11	9.2E+10	0	0	0	0	0
25	1.4E+12	6.5E+11	6.6E+11	9.0333E+11	4.3E+11	3E+10	2E+10	3.4E+11	1.3E+11	1.82E+11
12.5	8.9E+11	1.2E+12	1.5E+12	1.1967E+12	3.1E+11	4.3E+11	3.4E+11	3.1E+11	3.6E+11	6.24E+10
6.25	9E+11	1.1E+11	1.1E+12	7.0333E+11	5.2E+11	4.3E+11	4.1E+11	2.8E+11	3.73E+11	8.14E+10
3.125	1E+12	1.1E+12	1.2E+11	7.4E+11	5.4E+11	5.4E+11	5.2E+11	5.8E+11	5.47E+11	3.06E+10
1.562	1.2E+12	1.1E+12	1.2E+12	1.1667E+12	5.8E+10	6.3E+11	5E+11	6E+11	5.77E+11	6.81E+10
0.781	1.3E+12	1.1E+12	1.4E+12	1.2667E+12	1.5E+11	na	1.1E+12	1E+12	1.05E+12	7.07E+10
0.39	1.5E+12	1.3E+12	1.4E+12	1.4E+12	1E+11	1.2E+12	1.3E+12	1.2E+12	1.23E+12	5.77E+10
	acetone					aqueous extract (macerated)				
conc.	trial 1	trial 2	trial 3	average	STD	trial 1	trial 2	trial 3	average	STD
50	0	0	0	0	0	0	0	0	0	0
25	6.5E+11	8E+11	5E+11	6.5E+11	1.5E+11	0	0	0	0	0
12.5	6.4E+11	6.2E+11	8.3E+11	6.9667E+11	1.2E+11	1.2E+11	8E+10	6E+10	8.67E+10	3.06E+10
6.25	7.8E+11	7E+11	7.1E+11	7.3E+11	4.4E+10	3.5E+11	3.3E+11	3.1E+11	3.3E+11	2E+10
3.125	7.1E+11	7.2E+11	9E+11	7.7667E+11	1.1E+11	4.8E+11	3.4E+11	3.7E+11	3.97E+11	7.37E+10
1.562	8E+11	8E+11	9E+11	8.3333E+11	5.8E+10	5.4E+11	5.6E+11	4E+11	5E+11	8.72E+10
0.781	1.3E+12	1.4E+12	1.4E+12	1.3667E+12	5.8E+10	4.4E+11	5.8E+11	4.8E+11	5E+11	7.21E+10
0.39	1.5E+12	1.6E+12	1.5E+12	1.5333E+12	5.8E+10	5.5E+11	5E+11	5E+11	5.17E+11	2.89E+10
	aqueous extract (microwaved)					aqueous extract (decoction)				
conc.	trial 1	trial 2	trial 3	average	STD	trial 1	trial 2	trial 3	average	STD
50	0	0	0	0	0	0	0	0	0	0
25	0	0	0	0	0	3.8E+11	2.5E+11	1E+10	2.13E+11	1.88E+11
12.5	2.1E+11	1E+11	3E+11	2.0333E+11	1E+11	8E+10	1.3E+11	2E+11	1.37E+11	6.03E+10
6.25	6.2E+11	7E+11	6.1E+11	6.4333E+11	4.9E+10	3.5E+11	1.5E+11	3.5E+11	2.83E+11	1.15E+11
3.125	6.4E+11	7.1E+11	6.2E+11	6.5667E+11	4.7E+10	3.8E+11	2.6E+11	2.8E+11	3.07E+11	6.43E+10
1.562	6.6E+11	6.8E+11	6.3E+11	6.5667E+11	2.5E+10	2.8E+11	2E+11	4.6E+11	3.13E+11	1.33E+11
0.781	na	7E+11	8E+11	7.5E+11	7.1E+10	3.3E+11	4.3E+11	3.5E+11	3.7E+11	5.29E+10
0.39	8.4E+11	8.1E+11	8.7E+11	8.4E+11	3E+10	3.1E+11	4.5E+11	3.7E+11	3.77E+11	7.02E+10

aqueous extract (infusion)					
**conc.**	**trial 1**	**trial 2**	**trial 3**	**average**	**STD**
50	0	0	0	0	0
25	1.6E+11	1.1E+11		1.35E+11	3.5E+10
12.5	1.9E+11	2.1E+11	3E+11	2.3333E+11	1.4E+10
6.25	3.8E+11	4.2E+11	3.4E+11	3.8E+11	2.8E+10
3.125	4.4E+11	2.7E+11	4.3E+11	3.8E+11	1.2E+11
1.562	3.8E+11	4.9E+11	3.9E+11	4.2E+11	7.8E+10
0.781	5.6E+11	4.9E+11	3.9E+11	4.8E+11	4.9E+10
0.39	7E+11	6.6E+11	6.2E+11	6.6E+11	2.8E+10

Antimicrobial activity against *P.aeruginosa*										
	100% ethanol					50%ethanol				
**conc.**	**trial 1**	**trial 2**	**trial 3**	**average**	**STD**	**trial 1**	**trial 2**	**trial 3**	**average**	**STD**
**50**	0	0	0	0	0	0	0	0	0	0
**25**	3.9E+08	4.9E+08		4.4E+08	7.1E+07	5.3E+08	5.6E+08		5.45E+08	2.1E+07
**12.5**	4.6E+08	5.5E+08	4.6E+08	4.9E+08	5.2E+07	5.3E+08	5.8E+08		5.55E+08	3.5E+07
**6.25**	5.6E+08	4.6E+08	6.1E+08	5.4E+08	7.6E+07	4.6E+08	5.9E+08	5.1E+08	5.2E+08	6.6E+07
**3.125**	7.2E+08	5.4E+08	4.9E+08	5.8E+08	1.2E+08	7.5E+08	4.2E+08	5E+08	5.57E+08	1.7E+08
**1.562**	5.1E+08	5.6E+08	7E+08	5.9E+08	9.8E+07	6.8E+08	6.4E+08	5E+08	6.07E+08	9.5E+07
**0.781**	3.7E+08	7.2E+08	5.6E+08	5.5E+08	1.8E+08	6.8E+08	6.3E+08	4.8E+08	5.97E+08	1E+08
**0.39**	5.6E+08	7.8E+08	6.4E+08	6.6E+08	1.1E+08	6.4E+08	6.5E+08	6.2E+08	6.37E+08	1.5E+07
	acetone					aqueous extract (macerated)				
**conc.**	**trial 1**	**trial 2**	**trial 3**	**average**	**STD**	**trial 1**	**trial 2**	**trial 3**	**average**	**STD**
**50**	0	0	0	0	0	0	0	0	0	0
**25**	3.2E+08	5.7E+08	5.4E+08	4.8E+08	1.4E+08	0	0	0	0	0
**12.5**	5.9E+08	5.4E+08	4.6E+08	5.3E+08	6.6E+07	4.2E+08	4.7E+08	5E+08	4.63E+08	4E+07
**6.25**	8.3E+08	5.6E+08	7.8E+08	7.2E+08	1.4E+08	6.7E+08	7.8E+08	7.4E+08	7.3E+08	5.6E+07
**3.125**	6.8E+08	8.6E+08	7.1E+08	7.5E+08	9.6E+07	7.8E+08	7.9E+08	8.2E+08	7.97E+08	2.1E+07
**1.562**	8.1E+08	7.6E+08	7.9E+08	7.9E+08	2.5E+07	7.4E+08	8.4E+08	8.2E+08	8E+08	5.3E+07
**0.781**	8.1E+08	8.7E+08	7.7E+08	8.2E+08	5E+07	9E+08	8.5E+08	8.7E+08	8.73E+08	2.5E+07
**0.39**	7.9E+08	8.8E+08	8E+08	8.2E+08	4.9E+07	9.5E+08	9.3E+08	8.3E+08	9.03E+08	6.4E+07
	aqueous extract (microwaved)					aqueous extract (decoction)				
**conc.**	**trial 1**	**trial 2**	**trial 3**	**average**	**STD**	**trial 1**	**trial 2**	**trial 3**	**average**	**STD**
**50**	0	0	0	0	0	2.4E+08	2.5E+08		2.45E+08	7071068
**25**	0	0	0	0	0	3.4E+08	3.8E+08	5.4E+08	4.2E+08	1.1E+08
**12.5**	3.2E+08	3.4E+08	3.5E+08	3.4E+08	1.5E+07	4.6E+08	5.2E+08	3.7E+08	4.5E+08	7.5E+07
**6.25**	1.2E+09	8.6E+08	8.4E+08	9.7E+08	2E+08	5.1E+08	5.3E+08	5.8E+08	5.4E+08	3.6E+07
**3.125**	6.1E+08	1.1E+09	9.6E+08	8.9E+08	2.5E+08	5.6E+08	6.9E+08	3.6E+08	5.37E+08	1.7E+08
**1.562**	8.7E+08	9.6E+08	9.2E+08	9.2E+08	4.5E+07	6.8E+08	7.4E+08	6.8E+08	7E+08	3.5E+07
**0.781**	9.2E+08	9.2E+08	9.5E+08	9.3E+08	1.7E+07	6.5E+08	9.1E+08	6.2E+08	7.27E+08	1.6E+08
**0.39**	8E+08	9.7E+08	1.1E+09	9.6E+08	1.5E+08	8E+08	7.8E+08	7.4E+08	7.73E+08	3.1E+07

aqueous extract (infusion)					
**conc.**	**trial 1**	**trial 2**	**trial 3**	**average**	**STD**
50	0	0	0	0	0
25	3.8E+08	3.2E+08	3.1E+08	3.4E+08	3.8E+07
12.5	3.4E+08	3.2E+08	3.5E+08	3.4E+08	1.5E+07
6.25	6.6E+08	7.7E+08	7.6E+08	7.3E+08	6.1E+07
3.125	7.9E+08	7.7E+08	6.8E+08	7.5E+08	5.9E+07
1.562	8.7E+08	8E+08	6E+08	7.6E+08	1.4E+08
0.781	9.2E+08	8.1E+08	7.7E+08	8.3E+08	7.8E+07
0.39	8.6E+08	8.4E+08	8.6E+08	8.5E+08	1.2E+07

Antimicrobial activity against *P.mirabilis*										
	100% ethanol					50%ethanol				
**conc.**	**trial 1**	**trial 2**	**trial 3**	**average**	**STD**	**trial 1**	**trial 2**	**trial 3**	**average**	**STD**
**50**	0	0	0	0	0	0	0	0	0	0
**25**	0	0	0	0	0	0	0	0	0	0
**12.5**	2.72E+08	3.43E+08	7.3E+08	4.5E+08	2.5E+08	1.44E+09	5.83E+08	7.52E+08	9.24E+08	4.5E+08
**6.25**	4.67E+08	4.73E+08	7.1E+08	5.5E+08	1.4E+08	8.03E+08	6.32E+08	5.14E+08	6.49E+08	1.5E+08
**3.125**	6.92E+08	6.11E+08	4.9E+08	6E+08	1E+08	7.93E+08	6.26E+08	5.51E+08	6.57E+08	1.2E+08
**1.562**	6.13E+08	7.09E+08	7.1E+08	6.8E+08	5.6E+07	1.04E+09	1.05E+09	1.13E+09	1.07E+09	5.3E+07
**0.781**	7.78E+08	7.11E+08	8.2E+08	7.7E+08	5.4E+07	1.15E+09	1.04E+09	9.47E+08	1.05E+09	1E+08
**0.39**	1.12E+09	1.13E+09	8.2E+08	1E+09	1.8E+08	1.26E+09	1.28E+09	1.21E+09	1.25E+09	3.8E+07
	acetone					aqueous extract (macerated)				
**conc.**	**trial 1**	**trial 2**	**trial 3**	**average**	**STD**	**trial 1**	**trial 2**	**trial 3**	**average**	**STD**
**50**	0	0	0	0	0	1.61E+09	1.2E+09	7.43E+08	1.18E+09	4.4E+08
**25**	3.96E+08	6.08E+08	4.3E+08	4.8E+08	1.1E+08	6.28E+08	1.42E+09	1.25E+09	1.1E+09	4.2E+08
**12.5**	5.94E+08	7.28E+08	3.7E+08	5.6E+08	1.8E+08	9.77E+08	9.96E+08	1.26E+09	1.08E+09	1.6E+08
**6.25**	5.18E+08	8.16E+08	6E+08	6.4E+08	1.5E+08	9.86E+08	8.98E+08	8.16E+08	9E+08	8.5E+07
**3.125**	1.13E+09	1.13E+09	9.4E+08	1.1E+09	1.1E+08	1.1E+09	1.46E+09	9.11E+08	1.16E+09	2.8E+08
**1.562**	5.57E+08	1.12E+09	3.8E+08	6.8E+08	3.9E+08	1.13E+09	1.26E+09	8.23E+08	1.07E+09	2.2E+08
**0.781**	4.03E+08	1.1E+09	8.5E+08	7.8E+08	3.5E+08	6.98E+08	7.48E+08	8.06E+08	7.51E+08	5.4E+07
**0.39**	1.09E+09	1.07E+09	1.1E+09	1.1E+09	4.3E+07	1.06E+09	9.94E+08	1.01E+09	1.02E+09	3.5E+07
	aqueous extract (microwaved)					aqueous extract (decoction)				
**conc.**	**trial 1**	**trial 2**	**trial 3**	**average**	**STD**	**trial 1**	**trial 2**	**trial 3**	**average**	**STD**
**50**	0	0	0	0	0	5.64E+08	5.04E+08	6.75E+08	5.81E+08	8.7E+07
**25**	6.26E+08	3.84E+08	2.8E+08	4.3E+08	1.8E+08	8.51E+08	6.41E+08	1.39E+09	9.6E+08	3.8E+08
**12.5**	5.85E+08	9.99E+08	8E+08	7.9E+08	2.1E+08	1.01E+09	1.55E+09	1.04E+09	1.2E+09	3E+08
**6.25**	6.92E+08	9.24E+08	6.6E+08	7.6E+08	1.5E+08	1.27E+09	1.97E+09	1.03E+09	1.42E+09	4.9E+08
**3.125**	9.69E+08	6.13E+08	2.6E+08	6.1E+08	3.6E+08	1.06E+09	1.45E+09	1.11E+09	1.2E+09	2.1E+08
**1.562**	8.85E+08	9.32E+08	9.6E+08	9.3E+08	3.7E+07	7.71E+08	1.28E+09	1.01E+09	1.02E+09	2.5E+08
**0.781**	7.65E+08	9.49E+08	1E+09	9.1E+08	1.3E+08	8.06E+08	9.68E+08	8.74E+08	8.83E+08	8.1E+07
**0.39**	1.36E+09	1.04E+09	9.9E+08	1.1E+09	2E+08	9.3E+08	1.27E+09	9.45E+08	1.05E+09	1.9E+08

aqueous extract (infusion)					
**conc.**	**trial 1**	**trial 2**	**trial 3**	**average**	**STD**
50	7.61E+08	6.68E+08	5.2E+08	6.5E+08	1.2E+08
25	8.48E+08	8.48E+08	7.7E+08	8.2E+08	4.7E+07
12.5	1.34E+09	1.34E+09	1.4E+09	1.3E+09	1.1E+07
6.25	1.43E+09	1.34E+09	7.2E+08	1.2E+09	3.9E+08
3.125	1.44E+09	1.28E+09	1.4E+09	1.4E+09	7.8E+07
1.562	1.34E+09	1.39E+09	1.3E+09	1.3E+09	4.5E+07
0.781	1.22E+09	1.25E+09	1.2E+09	1.2E+09	2.1E+07
0.39	1.46E+09	1.48E+09	1.4E+09	1.5E+09	1.6E+07
