# Supplementary material for: Exploring the antimicrobial potential of pomegranate peel extracts (PPEs): Extraction techniques and bacterial susceptibility
Source: PLoS One. 2024 Dec 9;19(12):e0315173. doi: 10.1371/journal.pone.0315173 (PMC11627421; doi:10.1371/journal.pone.0315173)
Supplement: S2 File — (PDF) [file pone.0315173.s002.pdf]

**Supplementary 2: statistical analysis-Normality**

# Explore

## Extract Type

**Case Processing Summary**

| | | Cases | | | | | |
|---|---|---|---|---|---|---|---|
| | | Valid | | Missing | | Total | |
| | Extract Type | N | Percent | N | Percent | N | Percent |
| CFU_SAUREUS | Organic-PPEs | 27 | 100.0% | 0 | 0.0% | 27 | 100.0% |
| | Aqueous-PPEs | 36 | 100.0% | 0 | 0.0% | 36 | 100.0% |
| CFU_ECOLI | Organic-PPEs | 27 | 100.0% | 0 | 0.0% | 27 | 100.0% |
| | Aqueous-PPEs | 36 | 100.0% | 0 | 0.0% | 36 | 100.0% |
| CFU_PAERUGINOSA | Organic-PPEs | 27 | 100.0% | 0 | 0.0% | 27 | 100.0% |
| | Aqueous-PPEs | 36 | 100.0% | 0 | 0.0% | 36 | 100.0% |
| CFU_MIRABILIS | Organic-PPEs | 27 | 100.0% | 0 | 0.0% | 27 | 100.0% |
| | Aqueous-PPEs | 36 | 100.0% | 0 | 0.0% | 36 | 100.0% |

**Descriptives**

| | Extract Type | | | Statistic | Std. Error |
|---|---|---|---|---|---|
| CFU_SAUREUS | Organic-PPEs | Mean | | 633333333.33 | 127600025.007 |
| | | 95% Confidence Interval for Mean | Lower Bound | 371047725.56 | |
| | | | Upper Bound | 895618941.11 | |
| | | 5% Trimmed Mean | | 607880658.44 | |
| | | Median | | 590000000.00 | |
| | | Variance | | 439607692307692220.000 | |
| | | Std. Deviation | | 663029179.077 | |
| | | Minimum | | 0 | |
| | | Maximum | | 1730000000 | |
| | | Range | | 1730000000 | |
| | | Interquartile Range | | 1410000000 | |
| | | Skewness | | .394 | .448 |
| | | Kurtosis | | -1.535 | .872 |
| | Aqueous-PPEs | Mean | | 164451388.89 | 52896653.339 |
| | | 95% Confidence Interval for Mean | Lower Bound | 57065473.57 | |
| | | | Upper Bound | 271837304.21 | |
| | | 5% Trimmed Mean | | 126242283.95 | |
| | | Median | | .00 | |
| | | Variance | | 100730013640873040.000 | |
| | | Std. Deviation | | 317379920.034 | |
| | | Minimum | | 0 | |
| | | Maximum | | 1030000000 | |
| | | Range | | 1030000000 | |
| | | Interquartile Range | | 130000000 | |
| | | Skewness | | 2.083 | .393 |
| | | Kurtosis | | 2.950 | .768 |
| CFU_ECOLI | Organic-PPEs | Mean | | 542592592592.59 | 87426961934.539 |
| | | 95% Confidence Interval for Mean | Lower Bound | 362883898605.05 | |
| | | | Upper Bound | 722301286580.13 | |
| | | 5% Trimmed Mean | | 520987654320.99 | |
| | | Median | | 620000000000.00 | |
| | | Variance | | 206373789173789200000000.000 | |
| | | Std. Deviation | | 454283820066.035 | |
| | | Minimum | | 0 | |
| | | Maximum | | 1500000000000 | |
| | | Range | | 1500000000000 | |
| | | Interquartile Range | | 820000000000 | |
| | | Skewness | | .368 | .448 |
| | | Kurtosis | | -.695 | .872 |
| | Aqueous-PPEs | Mean | | 84027777777.78 | 18055051885.527 |
| | | 95% Confidence Interval for Mean | Lower Bound | 47374073800.00 | |
| | | | Upper Bound | 120681481755.55 | |
| | | 5% Trimmed Mean | | 74228395061.73 | |
| | | Median | | 5000000000.00 | |
| | | Variance | | 11735456349206350000000.000 | |
| | | Std. Deviation | | 108330311313.161 | |
| | | Minimum | | 0 | |
| | | Maximum | | 380000000000 | |
| | | Range | | 380000000000 | |
| | | Interquartile Range | | 153750000000 | |
| | | Skewness | | 1.119 | .393 |
| | | Kurtosis | | .313 | .768 |
| CFU_PAERUGINOSA | Organic-PPEs | Mean | | 337407407.41 | 48109095.493 |
| | | 95% Confidence Interval for Mean | Lower Bound | 238517745.36 | |
| | | | Upper Bound | 436297069.46 | |
| | | 5% Trimmed Mean | | 342263374.49 | |
| | | Median | | 460000000.00 | |
| | | Variance | | 62491096866096856.000 | |
| | | Std. Deviation | | 249982193.098 | |
| | | Minimum | | 0 | |
| | | Maximum | | 590000000 | |
| | | Range | | 590000000 | |
| | | Interquartile Range | | 545000000 | |
| | | Skewness | | -.589 | .448 |
| | | Kurtosis | | -1.603 | .872 |
| | Aqueous-PPEs | Mean | | 215694444.44 | 32695779.531 |
| | | 95% Confidence Interval for Mean | Lower Bound | 149318483.20 | |
| | | | Upper Bound | 282070405.69 | |
| | | 5% Trimmed Mean | | 210154320.99 | |
| | | Median | | 280000000.00 | |
| | | Variance | | 38484503968253976.000 | |
| | | Std. Deviation | | 196174677.184 | |

| | | | | | |
|---|---|---|---|---|---|
| | | Minimum | | 0 | |
| | | Maximum | | 540000000 | |
| | | Range | | 540000000 | |
| | | Interquartile Range | | 365000000 | |
| | | Skewness | | .007 | .393 |
| | | Kurtosis | | -1.628 | .768 |
| CFU_MIRABILIS | Organic-PPEs | Mean | | 268125000.00 | 70429656.669 |
| | | 95% Confidence Interval for Mean | Lower Bound | 123354767.36 | |
| | | | Upper Bound | 412895232.64 | |
| | | 5% Trimmed Mean | | 227982253.09 | |
| | | Median | | .00 | |
| | | Variance | | 133929086538461536.000 | |
| | | Std. Deviation | | 365963231.129 | |
| | | Minimum | | 0 | |
| | | Maximum | | 1436250000 | |
| | | Range | | 1436250000 | |
| | | Interquartile Range | | 583125000 | |
| | | Skewness | | 1.465 | .448 |
| | | Kurtosis | | 2.350 | .872 |
| | Aqueous-PPEs | Mean | | 844798611.11 | 70707562.585 |
| | | 95% Confidence Interval for Mean | Lower Bound | 701254627.72 | |
| | | | Upper Bound | 988342594.50 | |
| | | 5% Trimmed Mean | | 850748456.79 | |
| | | Median | | 823125000.00 | |
| | | Variance | | 179984138640872992.000 | |
| | | Std. Deviation | | 424245375.509 | |
| | | Minimum | | 0 | |
| | | Maximum | | 1612500000 | |
| | | Range | | 1612500000 | |
| | | Interquartile Range | | 643593750 | |
| | | Skewness | | -.206 | .393 |
| | | Kurtosis | | -.384 | .768 |

## Tests of Normality

| | | Kolmogorov-Smirnov[a] | | | Shapiro-Wilk | | |
|---|---|---|---|---|---|---|---|
| | Extract Type | Statistic | df | Sig. | Statistic | df | Sig. |
| CFU_SAUREUS | Organic-PPEs | .275 | 27 | .000 | .815 | 27 | .000 |
| | Aqueous-PPEs | .324 | 36 | .000 | .567 | 36 | .000 |
| CFU_ECOLI | Organic-PPEs | .167 | 27 | .052 | .919 | 27 | .037 |
| | Aqueous-PPEs | .281 | 36 | .000 | .787 | 36 | .000 |
| CFU_PAERUGINOSA | Organic-PPEs | .252 | 27 | .000 | .742 | 27 | .000 |
| | Aqueous-PPEs | .281 | 36 | .000 | .820 | 36 | .000 |
| CFU_MIRABILIS | Organic-PPEs | .324 | 27 | .000 | .753 | 27 | .000 |
| | Aqueous-PPEs | .082 | 36 | .200* | .967 | 36 | .343 |

*. This is a lower bound of the true significance.
a. Lilliefors Significance Correction

# SOLVENT

Descriptives

| | Solvent | | | Statistic | Std. Error |
|---|---|---|---|---|---|
| CFU_SAUREUS | 50%Eth | Mean | | 483333333.33 | 241827532.666 |
| | | 95% Confidence Interval for Mean | Lower Bound | -74321957.00 | |
| | | | Upper Bound | 1040988623.67 | |
| | | 5% Trimmed Mean | | 453148148.15 | |
| | | Median | | .00 | |
| | | Variance | | 526325000000000000.000 | |
| | | Std. Deviation | | 725482597.999 | |
| | | Minimum | | 0 | |
| | | Maximum | | 1510000000 | |
| | | Range | | 1510000000 | |
| | | Interquartile Range | | 1420000000 | |
| | | Skewness | | .862 | .717 |
| | | Kurtosis | | -1.691 | 1.400 |
| | 100%Eth | Mean | | 1243333333.33 | 128495568.449 |
| | | 95% Confidence Interval for Mean | Lower Bound | 947022021.13 | |
| | | | Upper Bound | 1539644645.53 | |
| | | 5% Trimmed Mean | | 1248148148.15 | |
| | | Median | | 1270000000.00 | |
| | | Variance | | 148600000000000000.000 | |
| | | Std. Deviation | | 385486705.348 | |
| | | Minimum | | 670000000 | |
| | | Maximum | | 1730000000 | |
| | | Range | | 1060000000 | |
| | | Interquartile Range | | 680000000 | |
| | | Skewness | | -.098 | .717 |
| | | Kurtosis | | -1.525 | 1.400 |
| | Acetone | Mean | | 173333333.33 | 89333955.222 |
| | | 95% Confidence Interval for Mean | Lower Bound | -32671136.82 | |
| | | | Upper Bound | 379337803.49 | |
| | | 5% Trimmed Mean | | 159259259.26 | |
| | | Median | | .00 | |
| | | Variance | | 71825000000000000.000 | |
| | | Std. Deviation | | 268001865.665 | |
| | | Minimum | | 0 | |
| | | Maximum | | 600000000 | |
| | | Range | | 600000000 | |
| | | Interquartile Range | | 480000000 | |
| | | Skewness | | 1.055 | .717 |
| | | Kurtosis | | -.927 | 1.400 |
| | macerate | Mean | | .00 | .000 |
| | | 95% Confidence Interval for Mean | Lower Bound | .00 | |
| | | | Upper Bound | .00 | |
| | | 5% Trimmed Mean | | .00 | |
| | | Median | | .00 | |
| | | Variance | | .000 | |
| | | Std. Deviation | | .000 | |
| | | Minimum | | 0 | |
| | | Maximum | | 0 | |
| | | Range | | 0 | |
| | | Interquartile Range | | 0 | |
| | | Skewness | | . | . |
| | | Kurtosis | | . | . |
| | MA_PPE | Mean | | .00 | .000 |
| | | 95% Confidence Interval for Mean | Lower Bound | .00 | |
| | | | Upper Bound | .00 | |
| | | 5% Trimmed Mean | | .00 | |
| | | Median | | .00 | |
| | | Variance | | .000 | |
| | | Std. Deviation | | .000 | |
| | | Minimum | | 0 | |
| | | Maximum | | 0 | |
| | | Range | | 0 | |
| | | Interquartile Range | | 0 | |
| | | Skewness | | . | . |
| | | Kurtosis | | . | . |
| | decoction | Mean | | 589333333.33 | 134190577.579 |
| | | 95% Confidence Interval for Mean | Lower Bound | 279889306.53 | |
| | | | Upper Bound | 898777360.14 | |
| | | 5% Trimmed Mean | | 590370370.37 | |
| | | Median | | 690000000.00 | |
| | | Variance | | 162064000000000000.000 | |
| | | Std. Deviation | | 402571732.738 | |
| | | Minimum | | 130000000 | |
| | | Maximum | | 1030000000 | |
| | | Range | | 900000000 | |
| | | Interquartile Range | | 827000000 | |
| | | Skewness | | -.107 | .717 |
| | | Kurtosis | | -2.305 | 1.400 |
| | infusion | Mean | | 68472222.22 | 21925316.910 |
| | | 95% Confidence Interval for Mean | Lower Bound | 17912350.76 | |
| | | | Upper Bound | 119032093.68 | |

| | | | | | |
|---|---|---|---|---|---|
| | | 5% Trimmed Mean | | 67746913.58 | |
| | | Median | | 68750000.00 | |
| | | Variance | | 4326475694444444.500 | |
| | | Std. Deviation | | 65775950.730 | |
| | | Minimum | | 0 | |
| | | Maximum | | 150000000 | |
| | | Range | | 150000000 | |
| | | Interquartile Range | | 130000000 | |
| | | Skewness | | .028 | .717 |
| | | Kurtosis | | -2.299 | 1.400 |
| CFU_ECOLI | 50%Eth | Mean | | 163333333333.33 | 61621605157.787 |
| | | 95% Confidence Interval for Mean | Lower Bound | 21233657021.87 | |
| | | | Upper Bound | 305433009644.80 | |
| | | 5% Trimmed Mean | | 157592592592.59 | |
| | | Median | | 30000000000.00 | |
| | | Variance | | 34175000000000000700 0000.000 | |
| | | Std. Deviation | | 184864815473.362 | |
| | | Minimum | | 0 | |
| | | Maximum | | 430000000000 | |
| | | Range | | 430000000000 | |
| | | Interquartile Range | | 340000000000 | |
| | | Skewness | | .375 | .717 |
| | | Kurtosis | | -2.200 | 1.400 |
| | 100%Eth | Mean | | 1015555555555.56 | 100251227633.258 |
| | | 95% Confidence Interval for Mean | Lower Bound | 784375810074.47 | |
| | | | Upper Bound | 1246735301036.64 | |
| | | 5% Trimmed Mean | | 1008950617283.95 | |
| | | Median | | 1000000000000.00 | |
| | | Variance | | 90452777777777777000 0000.000 | |
| | | Std. Deviation | | 300753682899.774 | |
| | | Minimum | | 650000000000 | |
| | | Maximum | | 1500000000000 | |
| | | Range | | 850000000000 | |
| | | Interquartile Range | | 550000000000 | |
| | | Skewness | | .447 | .717 |
| | | Kurtosis | | -.868 | 1.400 |
| | Acetone | Mean | | 448888888888.89 | 116779046403.935 |
| | | 95% Confidence Interval for Mean | Lower Bound | 179595924976.80 | |
| | | | Upper Bound | 718181852800.98 | |
| | | 5% Trimmed Mean | | 452654320987.65 | |
| | | Median | | 620000000000.00 | |
| | | Variance | | 12273611111111111100 00000.000 | |
| | | Std. Deviation | | 350337139211.804 | |
| | | Minimum | | 0 | |
| | | Maximum | | 830000000000 | |
| | | Range | | 830000000000 | |
| | | Interquartile Range | | 725000000000 | |
| | | Skewness | | -.570 | .717 |
| | | Kurtosis | | -1.692 | 1.400 |
| | macerate | Mean | | 28888888888.89 | 15315609724.545 |
| | | 95% Confidence Interval for Mean | Lower Bound | -6428970469.01 | |
| | | | Upper Bound | 64206748246.79 | |
| | | 5% Trimmed Mean | | 25432098765.43 | |
| | | Median | | .00 | |
| | | Variance | | 21111111111111111300 000.000 | |
| | | Std. Deviation | | 45946829173.634 | |
| | | Minimum | | 0 | |
| | | Maximum | | 120000000000 | |
| | | Range | | 120000000000 | |
| | | Interquartile Range | | 70000000000 | |
| | | Skewness | | 1.285 | .717 |
| | | Kurtosis | | .288 | 1.400 |
| | MA_PPE | Mean | | 67777777777.78 | 37777777777.778 |
| | | 95% Confidence Interval for Mean | Lower Bound | -19337933996.41 | |
| | | | Upper Bound | 154893489551.97 | |
| | | 5% Trimmed Mean | | 58641975308.64 | |
| | | Median | | .00 | |
| | | Variance | | 12844444444444444500 0000.000 | |
| | | Std. Deviation | | 113333333333.333 | |
| | | Minimum | | 0 | |
| | | Maximum | | 300000000000 | |
| | | Range | | 300000000000 | |
| | | Interquartile Range | | 155000000000 | |
| | | Skewness | | 1.498 | .717 |
| | | Kurtosis | | 1.022 | 1.400 |
| | decoction | Mean | | 116666666666.67 | 45307590730.227 |
| | | 95% Confidence Interval for Mean | Lower Bound | 12187175086.85 | |
| | | | Upper Bound | 221146158246.48 | |
| | | 5% Trimmed Mean | | 108518518518.52 | |

| | | | | | |
|---|---|---|---|---|---|
| | | Median | | 80000000000.00 | |
| | | Variance | | 18475000000000000000000.000 | |
| | | Std. Deviation | | 135922772190.682 | |
| | | Minimum | | 0 | |
| | | Maximum | | 380000000000 | |
| | | Range | | 380000000000 | |
| | | Interquartile Range | | 225000000000 | |
| | | Skewness | | .972 | .717 |
| | | Kurtosis | | .027 | 1.400 |
| | infusion | Mean | | 122777777777.78 | 35444705676.502 |
| | | 95% Confidence Interval for Mean | Lower Bound | 41042139916.85 | |
| | | | Upper Bound | 204513415638.71 | |
| | | 5% Trimmed Mean | | 119753086419.75 | |
| | | Median | | 135000000000.00 | |
| | | Variance | | 11306944444444443000000.000 | |
| | | Std. Deviation | | 106334117029.505 | |
| | | Minimum | | 0 | |
| | | Maximum | | 300000000000 | |
| | | Range | | 300000000000 | |
| | | Interquartile Range | | 200000000000 | |
| | | Skewness | | .138 | .717 |
| | | Kurtosis | | -.908 | 1.400 |
| CFU_PAERUGINOSA | 50%Eth | Mean | | 366666666.67 | 91806711.205 |
| | | 95% Confidence Interval for Mean | Lower Bound | 154960010.99 | |
| | | | Upper Bound | 578373322.34 | |
| | | 5% Trimmed Mean | | 375185185.19 | |
| | | Median | | 530000000.00 | |
| | | Variance | | 75856250000000000.000 | |
| | | Std. Deviation | | 275420133.614 | |
| | | Minimum | | 0 | |
| | | Maximum | | 580000000 | |
| | | Range | | 580000000 | |
| | | Interquartile Range | | 557500000 | |
| | | Skewness | | -.845 | .717 |
| | | Kurtosis | | -1.714 | 1.400 |
| | 100%Eth | Mean | | 310000000.00 | 78757715.671 |
| | | 95% Confidence Interval for Mean | Lower Bound | 128384381.98 | |
| | | | Upper Bound | 491615618.02 | |
| | | 5% Trimmed Mean | | 313888888.89 | |
| | | Median | | 440000000.00 | |
| | | Variance | | 55825000000000000.000 | |
| | | Std. Deviation | | 236273147.014 | |
| | | Minimum | | 0 | |
| | | Maximum | | 550000000 | |
| | | Range | | 550000000 | |
| | | Interquartile Range | | 475000000 | |
| | | Skewness | | -.734 | .717 |
| | | Kurtosis | | -1.700 | 1.400 |
| | Acetone | Mean | | 335555555.56 | 87941127.894 |
| | | 95% Confidence Interval for Mean | Lower Bound | 132762950.98 | |
| | | | Upper Bound | 538348160.13 | |
| | | 5% Trimmed Mean | | 340061728.40 | |
| | | Median | | 460000000.00 | |
| | | Variance | | 69602777777777776.000 | |
| | | Std. Deviation | | 263823383.683 | |
| | | Minimum | | 0 | |
| | | Maximum | | 590000000 | |
| | | Range | | 590000000 | |
| | | Interquartile Range | | 555000000 | |
| | | Skewness | | -.569 | .717 |
| | | Kurtosis | | -1.863 | 1.400 |
| | macerate | Mean | | 154444444.44 | 77515430.563 |
| | | 95% Confidence Interval for Mean | Lower Bound | -24306458.97 | |
| | | | Upper Bound | 333195347.86 | |
| | | 5% Trimmed Mean | | 143827160.49 | |
| | | Median | | .00 | |
| | | Variance | | 54077777777777784.000 | |
| | | Std. Deviation | | 232546291.688 | |
| | | Minimum | | 0 | |
| | | Maximum | | 500000000 | |
| | | Range | | 500000000 | |
| | | Interquartile Range | | 445000000 | |
| | | Skewness | | .886 | .717 |
| | | Kurtosis | | -1.589 | 1.400 |
| | MA_PPE | Mean | | 112222222.22 | 56168837.193 |
| | | 95% Confidence Interval for Mean | Lower Bound | -17303348.61 | |
| | | | Upper Bound | 241747793.06 | |
| | | 5% Trimmed Mean | | 105246913.58 | |

| | | | | | |
|---|---|---|---|---|---|
| | | Median | | .00 | |
| | | Variance | | 28394444444444448.000 | |
| | | Std. Deviation | | 168506511.579 | |
| | | Minimum | | 0 | |
| | | Maximum | | 350000000 | |
| | | Range | | 350000000 | |
| | | Interquartile Range | | 330000000 | |
| | | Skewness | | .865 | .717 |
| | | Kurtosis | | -1.680 | 1.400 |
| | decoction | Mean | | 371666666.67 | 38622100.754 |
| | | 95% Confidence Interval for Mean | Lower Bound | 282603942.62 | |
| | | | Upper Bound | 460729390.72 | |
| | | 5% Trimmed Mean | | 369629629.63 | |
| | | Median | | 370000000.00 | |
| | | Variance | | 13425000000000000.000 | |
| | | Std. Deviation | | 115866302.263 | |
| | | Minimum | | 240000000 | |
| | | Maximum | | 540000000 | |
| | | Range | | 300000000 | |
| | | Interquartile Range | | 242500000 | |
| | | Skewness | | .266 | .717 |
| | | Kurtosis | | -1.412 | 1.400 |
| | infusion | Mean | | 224444444.44 | 56522146.867 |
| | | 95% Confidence Interval for Mean | Lower Bound | 94104140.04 | |
| | | | Upper Bound | 354784748.85 | |
| | | 5% Trimmed Mean | | 228271604.94 | |
| | | Median | | 320000000.00 | |
| | | Variance | | 28752777777777780.000 | |
| | | Std. Deviation | | 169566440.600 | |
| | | Minimum | | 0 | |
| | | Maximum | | 380000000 | |
| | | Range | | 380000000 | |
| | | Interquartile Range | | 345000000 | |
| | | Skewness | | -.800 | .717 |
| | | Kurtosis | | -1.705 | 1.400 |
| CFU_MIRABILIS | 50%Eth | Mean | | 307916666.67 | 171385318.850 |
| | | 95% Confidence Interval for Mean | Lower Bound | -87298587.31 | |
| | | | Upper Bound | 703131920.65 | |
| | | 5% Trimmed Mean | | 262337962.96 | |
| | | Median | | .00 | |
| | | Variance | | 264356347656249984.000 | |
| | | Std. Deviation | | 514155956.550 | |
| | | Minimum | | 0 | |
| | | Maximum | | 1436250000 | |
| | | Range | | 1436250000 | |
| | | Interquartile Range | | 667500000 | |
| | | Skewness | | 1.620 | .717 |
| | | Kurtosis | | 2.019 | 1.400 |
| | 100%Eth | Mean | | 149791666.67 | 85568779.698 |
| | | 95% Confidence Interval for Mean | Lower Bound | -47530293.16 | |
| | | | Upper Bound | 347113626.49 | |
| | | 5% Trimmed Mean | | 125706018.52 | |
| | | Median | | .00 | |
| | | Variance | | 65898144531250016.000 | |
| | | Std. Deviation | | 256706339.094 | |
| | | Minimum | | 0 | |
| | | Maximum | | 733125000 | |
| | | Range | | 733125000 | |
| | | Interquartile Range | | 307500000 | |
| | | Skewness | | 1.780 | .717 |
| | | Kurtosis | | 2.857 | 1.400 |
| | Acetone | Mean | | 346666666.67 | 94598991.454 |
| | | 95% Confidence Interval for Mean | Lower Bound | 128521001.19 | |
| | | | Upper Bound | 564812332.14 | |
| | | 5% Trimmed Mean | | 344768518.52 | |
| | | Median | | 395625000.00 | |
| | | Variance | | 80540722656250016.000 | |
| | | Std. Deviation | | 283796974.361 | |
| | | Minimum | | 0 | |
| | | Maximum | | 727500000 | |
| | | Range | | 727500000 | |
| | | Interquartile Range | | 600937500 | |
| | | Skewness | | -.254 | .717 |
| | | Kurtosis | | -1.565 | 1.400 |
| | macerate | Mean | | 1120416666.67 | 105100450.050 |
| | | 95% Confidence Interval for Mean | Lower Bound | 878054594.24 | |
| | | | Upper Bound | 1362778739.09 | |
| | | 5% Trimmed Mean | | 1120428240.74 | |

| | | | | | |
|---|---|---|---|---|---|
| | | Median | | 1198125000.00 | |
| | | Variance | | 99414941406250000.000 | |
| | | Std. Deviation | | 315301350.150 | |
| | | Minimum | | 628125000 | |
| | | Maximum | | 1612500000 | |
| | | Range | | 984375000 | |
| | | Interquartile Range | | 479062500 | |
| | | Skewness | | -.138 | .717 |
| | | Kurtosis | | -.584 | 1.400 |
| | MA_PPE | Mean | | 408541666.67 | 123429764.174 |
| | | 95% Confidence Interval for Mean | Lower Bound | 123912120.07 | |
| | | | Upper Bound | 693171213.26 | |
| | | 5% Trimmed Mean | | 398414351.85 | |
| | | Median | | 384375000.00 | |
| | | Variance | | 137114160156249984.000 | |
| | | Std. Deviation | | 370289292.522 | |
| | | Minimum | | 0 | |
| | | Maximum | | 999375000 | |
| | | Range | | 999375000 | |
| | | Interquartile Range | | 712500000 | |
| | | Skewness | | .237 | .717 |
| | | Kurtosis | | -1.265 | 1.400 |
| | decoction | Mean | | 912736111.11 | 122034570.474 |
| | | 95% Confidence Interval for Mean | Lower Bound | 631323886.96 | |
| | | | Upper Bound | 1194148335.26 | |
| | | 5% Trimmed Mean | | 900297067.90 | |
| | | Median | | 851250000.00 | |
| | | Variance | | 134031927517361120.000 | |
| | | Std. Deviation | | 366103711.423 | |
| | | Minimum | | 504375000 | |
| | | Maximum | | 1545000000 | |
| | | Range | | 1040625000 | |
| | | Interquartile Range | | 610437500 | |
| | | Skewness | | .716 | .717 |
| | | Kurtosis | | -.662 | 1.400 |
| | infusion | Mean | | 937500000.00 | 106686602.955 |
| | | 95% Confidence Interval for Mean | Lower Bound | 691480252.42 | |
| | | | Upper Bound | 1183519747.58 | |
| | | 5% Trimmed Mean | | 937604166.67 | |
| | | Median | | 847500000.00 | |
| | | Variance | | 102438281250000000.000 | |
| | | Std. Deviation | | 320059808.864 | |
| | | Minimum | | 517500000 | |
| | | Maximum | | 1355625000 | |
| | | Range | | 838125000 | |
| | | Interquartile Range | | 622500000 | |
| | | Skewness | | .465 | .717 |
| | | Kurtosis | | -1.513 | 1.400 |

Case Processing Summary

| | Solvent | Cases | | | | | |
|---|---|---|---|---|---|---|---|
| | | Valid | | Missing | | Total | |
| | | N | Percent | N | Percent | N | Percent |
| CFU_SAUREUS | 50%Eth | 9 | 100.0% | 0 | 0.0% | 9 | 100.0% |
| | 100%Eth | 9 | 100.0% | 0 | 0.0% | 9 | 100.0% |
| | Acetone | 9 | 100.0% | 0 | 0.0% | 9 | 100.0% |
| | macerate | 9 | 100.0% | 0 | 0.0% | 9 | 100.0% |
| | MA_PPE | 9 | 100.0% | 0 | 0.0% | 9 | 100.0% |
| | decoction | 9 | 100.0% | 0 | 0.0% | 9 | 100.0% |
| | infusion | 9 | 100.0% | 0 | 0.0% | 9 | 100.0% |
| CFU_ECOLI | 50%Eth | 9 | 100.0% | 0 | 0.0% | 9 | 100.0% |
| | 100%Eth | 9 | 100.0% | 0 | 0.0% | 9 | 100.0% |
| | Acetone | 9 | 100.0% | 0 | 0.0% | 9 | 100.0% |
| | macerate | 9 | 100.0% | 0 | 0.0% | 9 | 100.0% |
| | MA_PPE | 9 | 100.0% | 0 | 0.0% | 9 | 100.0% |
| | decoction | 9 | 100.0% | 0 | 0.0% | 9 | 100.0% |
| | infusion | 9 | 100.0% | 0 | 0.0% | 9 | 100.0% |
| CFU_PAERUGINOSA | 50%Eth | 9 | 100.0% | 0 | 0.0% | 9 | 100.0% |
| | 100%Eth | 9 | 100.0% | 0 | 0.0% | 9 | 100.0% |
| | Acetone | 9 | 100.0% | 0 | 0.0% | 9 | 100.0% |
| | macerate | 9 | 100.0% | 0 | 0.0% | 9 | 100.0% |
| | MA_PPE | 9 | 100.0% | 0 | 0.0% | 9 | 100.0% |
| | decoction | 9 | 100.0% | 0 | 0.0% | 9 | 100.0% |
| | infusion | 9 | 100.0% | 0 | 0.0% | 9 | 100.0% |
| CFU_MIRABILIS | 50%Eth | 9 | 100.0% | 0 | 0.0% | 9 | 100.0% |
| | 100%Eth | 9 | 100.0% | 0 | 0.0% | 9 | 100.0% |
| | Acetone | 9 | 100.0% | 0 | 0.0% | 9 | 100.0% |
| | macerate | 9 | 100.0% | 0 | 0.0% | 9 | 100.0% |
| | MA_PPE | 9 | 100.0% | 0 | 0.0% | 9 | 100.0% |
| | decoction | 9 | 100.0% | 0 | 0.0% | 9 | 100.0% |
| | infusion | 9 | 100.0% | 0 | 0.0% | 9 | 100.0% |

## Tests of Normality

| | Solvent | Kolmogorov-Smirnov[a] | | | Shapiro-Wilk | | |
|---|---|---|---|---|---|---|---|
| | | Statistic | df | Sig. | Statistic | df | Sig. |
| CFU_SAUREUS | 50%Eth | .414 | 9 | .000 | .633 | 9 | .000 |
| | 100%Eth | .189 | 9 | .200* | .927 | 9 | .449 |
| | Acetone | .408 | 9 | .000 | .664 | 9 | .001 |
| | macerate | . | 9 | . | . | 9 | . |
| | MA_PPE | . | 9 | . | . | 9 | . |
| | decoction | .245 | 9 | .128 | .812 | 9 | .028 |
| | infusion | .249 | 9 | .113 | .805 | 9 | .023 |
| CFU_ECOLI | 50%Eth | .320 | 9 | .008 | .768 | 9 | .009 |
| | 100%Eth | .187 | 9 | .200* | .934 | 9 | .521 |
| | Acetone | .243 | 9 | .134 | .807 | 9 | .025 |
| | macerate | .402 | 9 | .000 | .697 | 9 | .001 |
| | MA_PPE | .392 | 9 | .000 | .681 | 9 | .001 |
| | decoction | .228 | 9 | .195 | .857 | 9 | .088 |
| | infusion | .209 | 9 | .200* | .911 | 9 | .325 |
| CFU_PAERUGINOSA | 50%Eth | .390 | 9 | .000 | .663 | 9 | .001 |
| | 100%Eth | .299 | 9 | .020 | .754 | 9 | .006 |
| | Acetone | .237 | 9 | .155 | .782 | 9 | .013 |
| | macerate | .413 | 9 | .000 | .649 | 9 | .000 |
| | MA_PPE | .414 | 9 | .000 | .635 | 9 | .000 |
| | decoction | .186 | 9 | .200* | .902 | 9 | .262 |
| | infusion | .360 | 9 | .001 | .715 | 9 | .002 |
| CFU_MIRABILIS | 50%Eth | .392 | 9 | .000 | .686 | 9 | .001 |
| | 100%Eth | .387 | 9 | .000 | .675 | 9 | .001 |
| | Acetone | .222 | 9 | .200* | .870 | 9 | .123 |
| | macerate | .153 | 9 | .200* | .972 | 9 | .909 |
| | MA_PPE | .198 | 9 | .200* | .912 | 9 | .329 |
| | decoction | .186 | 9 | .200* | .914 | 9 | .344 |
| | infusion | .277 | 9 | .044 | .845 | 9 | .066 |

*. This is a lower bound of the true significance.
a. Lilliefors Significance Correction

# Concentration Level

**Case Processing Summary**

| | Concentration Level | Cases Valid N | Percent | Missing N | Percent | Total N | Percent |
|---|---|---|---|---|---|---|---|
| CFU_SAUREUS | 50 | 21 | 100.0% | 0 | 0.0% | 21 | 100.0% |
| | 25 | 21 | 100.0% | 0 | 0.0% | 21 | 100.0% |
| | 12.5 | 21 | 100.0% | 0 | 0.0% | 21 | 100.0% |
| CFU_ECOLI | 50 | 21 | 100.0% | 0 | 0.0% | 21 | 100.0% |
| | 25 | 21 | 100.0% | 0 | 0.0% | 21 | 100.0% |
| | 12.5 | 21 | 100.0% | 0 | 0.0% | 21 | 100.0% |
| CFU_PAERUGINOSA | 50 | 21 | 100.0% | 0 | 0.0% | 21 | 100.0% |
| | 25 | 21 | 100.0% | 0 | 0.0% | 21 | 100.0% |
| | 12.5 | 21 | 100.0% | 0 | 0.0% | 21 | 100.0% |
| CFU_MIRABILIS | 50 | 21 | 100.0% | 0 | 0.0% | 21 | 100.0% |
| | 25 | 21 | 100.0% | 0 | 0.0% | 21 | 100.0% |
| | 12.5 | 21 | 100.0% | 0 | 0.0% | 21 | 100.0% |

**Descriptives**

| | Concentration Level | | | Statistic | Std. Error |
|---|---|---|---|---|---|
| CFU_SAUREUS | 50 | Mean | | 174285714.29 | 86016530.606 |
| | | 95% Confidence Interval for Mean | Lower Bound | -5141624.42 | |
| | | | Upper Bound | 353713052.99 | |
| | | 5% Trimmed Mean | | 112195767.20 | |
| | | Median | | .00 | |
| | | Variance | | 155375714285714272.000 | |
| | | Std. Deviation | | 394177262.517 | |
| | | Minimum | | 0 | |
| | | Maximum | | 1490000000 | |
| | | Range | | 1490000000 | |
| | | Interquartile Range | | 130000000 | |
| | | Skewness | | 2.583 | .501 |
| | | Kurtosis | | 6.322 | .972 |
| | 25 | Mean | | 260297619.05 | 98819893.317 |
| | | 95% Confidence Interval for Mean | Lower Bound | 54162933.73 | |
| | | | Upper Bound | 466432304.37 | |
| | | 5% Trimmed Mean | | 207658730.16 | |
| | | Median | | .00 | |
| | | Variance | | 205072797619047616.000 | |
| | | Std. Deviation | | 452849641.293 | |
| | | Minimum | | 0 | |
| | | Maximum | | 1490000000 | |
| | | Range | | 1490000000 | |
| | | Interquartile Range | | 450000000 | |
| | | Skewness | | 1.638 | .501 |
| | | Kurtosis | | 1.529 | .972 |
| | 12.5 | Mean | | 661619047.62 | 140549101.337 |
| | | 95% Confidence Interval for Mean | Lower Bound | 368438759.68 | |
| | | | Upper Bound | 954799335.55 | |
| | | 5% Trimmed Mean | | 639074074.07 | |
| | | Median | | 590000000.00 | |
| | | Variance | | 414835047619047550.000 | |
| | | Std. Deviation | | 644076895.735 | |
| | | Minimum | | 0 | |
| | | Maximum | | 1730000000 | |
| | | Range | | 1730000000 | |
| | | Interquartile Range | | 1340000000 | |
| | | Skewness | | .407 | .501 |
| | | Kurtosis | | -1.452 | .972 |
| CFU_ECOLI | 50 | Mean | | 135238095238.10 | 74346739281.043 |
| | | 95% Confidence Interval for Mean | Lower Bound | -19846485325.56 | |
| | | | Upper Bound | 290322675801.75 | |
| | | 5% Trimmed Mean | | 94708994708.99 | |
| | | Median | | .00 | |
| | | Variance | | 116076190476190480000000.000 | |
| | | Std. Deviation | | 340699560428.525 | |
| | | Minimum | | 0 | |
| | | Maximum | | 1000000000000 | |
| | | Range | | 1000000000000 | |
| | | Interquartile Range | | 0 | |
| | | Skewness | | 2.234 | .501 |
| | | Kurtosis | | 3.365 | .972 |
| | 25 | Mean | | 290238095238.10 | 80791085810.783 |
| | | 95% Confidence Interval for Mean | Lower Bound | 121710843371.89 | |
| | | | Upper Bound | 458765347104.30 | |
| | | 5% Trimmed Mean | | 246296296296.30 | |
| | | Median | | 135000000000.00 | |
| | | Variance | | 137071190476190480000000.000 | |
| | | Std. Deviation | | 370231266205.585 | |
| | | Minimum | | 0 | |
| | | Maximum | | 1400000000000 | |
| | | Range | | 1400000000000 | |
| | | Interquartile Range | | 575000000000 | |
| | | Skewness | | 1.563 | .501 |
| | | Kurtosis | | 2.599 | .972 |
| | 12.5 | Mean | | 416190476190.48 | 86273309315.377 |
| | | 95% Confidence Interval for Mean | Lower Bound | 236227506483.95 | |
| | | | Upper Bound | 596153445897.01 | |
| | | 5% Trimmed Mean | | 376507936507.94 | |
| | | Median | | 300000000000.00 | |
| | | Variance | | 156304761904761930000000.000 | |
| | | Std. Deviation | | 395353970392.055 | |

| | | | | | |
|---|---|---|---|---|---|
| | | Minimum | | 60000000000 | |
| | | Maximum | | 1500000000000 | |
| | | Range | | 1440000000000 | |
| | | Interquartile Range | | 505000000000 | |
| | | Skewness | | 1.525 | .501 |
| | | Kurtosis | | 1.789 | .972 |
| CFU_PAERUGINOSA | 50 | Mean | | 35000000.00 | 19173394.264 |
| | | 95% Confidence Interval for Mean | Lower Bound | -4994999.60 | |
| | | | Upper Bound | 74994999.60 | |
| | | 5% Trimmed Mean | | 25013227.51 | |
| | | Median | | .00 | |
| | | Variance | | 7720000000000000.000 | |
| | | Std. Deviation | | 87863530.546 | |
| | | Minimum | | 0 | |
| | | Maximum | | 250000000 | |
| | | Range | | 250000000 | |
| | | Interquartile Range | | 0 | |
| | | Skewness | | 2.203 | .501 |
| | | Kurtosis | | 3.149 | .972 |
| | 25 | Mean | | 316904761.90 | 48347054.116 |
| | | 95% Confidence Interval for Mean | Lower Bound | 216054574.24 | |
| | | | Upper Bound | 417754949.57 | |
| | | 5% Trimmed Mean | | 320476190.48 | |
| | | Median | | 380000000.00 | |
| | | Variance | | 49086190476190472.000 | |
| | | Std. Deviation | | 221554035.116 | |
| | | Minimum | | 0 | |
| | | Maximum | | 570000000 | |
| | | Range | | 570000000 | |
| | | Interquartile Range | | 535000000 | |
| | | Skewness | | -.552 | .501 |
| | | Kurtosis | | -1.294 | .972 |
| | 12.5 | Mean | | 451666666.67 | 19848633.554 |
| | | 95% Confidence Interval for Mean | Lower Bound | 410263142.59 | |
| | | | Upper Bound | 493070190.74 | |
| | | 5% Trimmed Mean | | 451322751.32 | |
| | | Median | | 460000000.00 | |
| | | Variance | | 8273333333333333.000 | |
| | | Std. Deviation | | 90957865.703 | |
| | | Minimum | | 320000000 | |
| | | Maximum | | 590000000 | |
| | | Range | | 270000000 | |
| | | Interquartile Range | | 185000000 | |
| | | Skewness | | -.118 | .501 |
| | | Kurtosis | | -1.395 | .972 |
| CFU_MIRABILIS | 50 | Mean | | 344910714.29 | 102471098.620 |
| | | 95% Confidence Interval for Mean | Lower Bound | 131159748.16 | |
| | | | Upper Bound | 558661680.41 | |
| | | 5% Trimmed Mean | | 294747023.81 | |
| | | Median | | .00 | |
| | | Variance | | 220506847098214304.000 | |
| | | Std. Deviation | | 469581565.969 | |
| | | Minimum | | 0 | |
| | | Maximum | | 1612500000 | |
| | | Range | | 1612500000 | |
| | | Interquartile Range | | 671250000 | |
| | | Skewness | | 1.275 | .501 |
| | | Kurtosis | | 1.163 | .972 |
| | 25 | Mean | | 541261904.76 | 100479585.445 |
| | | 95% Confidence Interval for Mean | Lower Bound | 331665162.33 | |
| | | | Upper Bound | 750858647.20 | |
| | | 5% Trimmed Mean | | 522631613.76 | |
| | | Median | | 607500000.00 | |
| | | Variance | | 212019088913690464.000 | |
| | | Std. Deviation | | 460455306.098 | |
| | | Minimum | | 0 | |
| | | Maximum | | 1419375000 | |
| | | Range | | 1419375000 | |
| | | Interquartile Range | | 847500000 | |
| | | Skewness | | .435 | .501 |
| | | Kurtosis | | -.633 | .972 |
| | 12.5 | Mean | | 906785714.29 | 82329722.428 |
| | | 95% Confidence Interval for Mean | Lower Bound | 735048922.68 | |
| | | | Upper Bound | 1078522505.90 | |
| | | 5% Trimmed Mean | | 906701388.89 | |
| | | Median | | 976875000.00 | |
| | | Variance | | 142341847098214304.000 | |
| | | Std. Deviation | | 377282184.973 | |
| | | Minimum | | 271875000 | |
| | | Maximum | | 1545000000 | |
| | | Range | | 1273125000 | |
| | | Interquartile Range | | 707812500 | |
| | | Skewness | | .001 | .501 |
| | | Kurtosis | | -1.004 | .972 |

## Tests of Normality

| | | Kolmogorov-Smirnov[a] | | | Shapiro-Wilk | | |
|---|---|---|---|---|---|---|---|
| | Concentration Level | Statistic | df | Sig. | Statistic | df | Sig. |
| CFU_SAUREUS | 50 | .385 | 21 | .000 | .524 | 21 | .000 |
| | 25 | .332 | 21 | .000 | .643 | 21 | .000 |
| | 12.5 | .215 | 21 | .012 | .858 | 21 | .006 |
| CFU_ECOLI | 50 | .511 | 21 | .000 | .431 | 21 | .000 |
| | 25 | .217 | 21 | .011 | .793 | 21 | .001 |
| | 12.5 | .243 | 21 | .002 | .812 | 21 | .001 |
| CFU_PAERUGINOSA | 50 | .512 | 21 | .000 | .425 | 21 | .000 |
| | 25 | .209 | 21 | .017 | .822 | 21 | .001 |
| | 12.5 | .156 | 21 | .200* | .915 | 21 | .070 |
| CFU_MIRABILIS | 50 | .340 | 21 | .000 | .755 | 21 | .000 |
| | 25 | .166 | 21 | .136 | .905 | 21 | .044 |
| | 12.5 | .111 | 21 | .200* | .956 | 21 | .449 |

*. This is a lower bound of the true significance.
a. Lilliefors Significance Correction

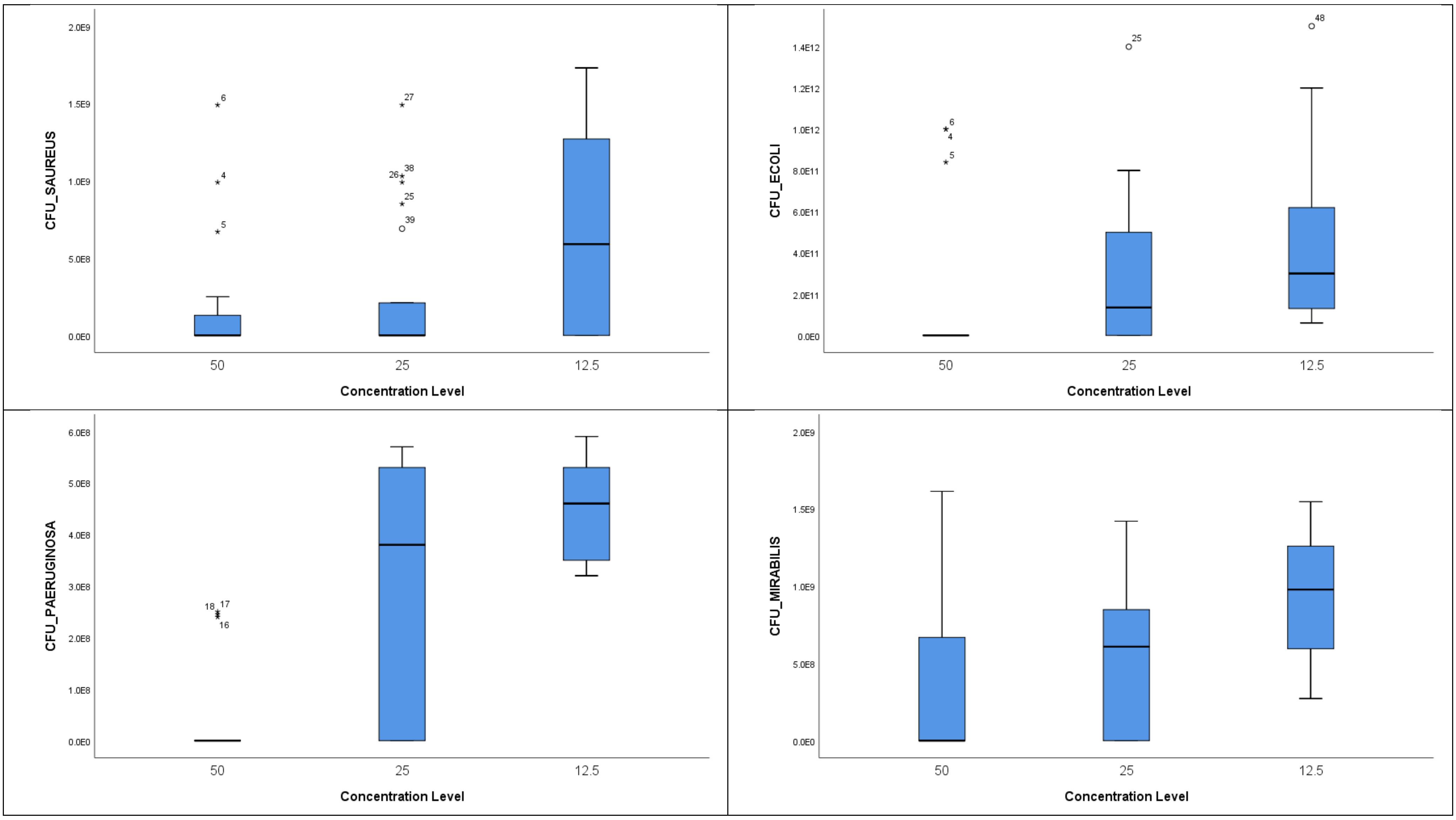

CFU_SAUREUS
2.0E9
1.5E9
1.0E9
5.0E8
0.0E0
6
4
5
27
38
26
25
39
CFU_ECOLI
1.4E12
1.2E12
1.0E12
8.0E11
6.0E11
4.0E11
2.0E11
0.0E0
6
4
5
25
48
CFU_PAERUGINOSA
6.0E8
5.0E8
4.0E8
3.0E8
2.0E8
1.0E8
0.0E0
18
17
16
CFU_MIRABILIS
2.0E9
1.5E9
1.0E9
5.0E8
0.0E0
50
25
12.5
Concentration Level
