# Supplementary material for: Exploring the antimicrobial potential of pomegranate peel extracts (PPEs): Extraction techniques and bacterial susceptibility
Source: PLoS One. 2024 Dec 9;19(12):e0315173. doi: 10.1371/journal.pone.0315173 (PMC11627421; doi:10.1371/journal.pone.0315173)
Supplement: S3 File — (PDF) [file pone.0315173.s003.pdf]

Supplementary 3: statistical analysis- non-parametric analyses

NON PARAMETRIC TESTS

EXTRACT TYPES: Aqueous versus organic

Descriptive Statistics

	N	Mean	Std. Deviation	Minimum	Maximum	Percentiles 25th	50th (Median)	75th
CFU_SAUREUS	63	365400793.65	543989536.671	0	1730000000	.00	.00	690000000.00
CFU_ECOLI	63	280555555555.56	381440511481.519	0	1500000000000	.00	110000000000.00	430000000000.00
CFU_PAERUGINOSA	62	263225806.45	226031509.435	0	590000000	.00	320000000.00	462500000.00
CFU_MIRABILIS	63	597652777.78	490431186.007	0	1612500000	.00	607500000.00	976875000.00
Extract Type	63	1.57	.499	1	2	1.00	2.00	2.00

Mann-Whitney Test

Ranks

	Extract Type	N	Mean Rank	Sum of Ranks
**CFU_SAUREUS**	**Organic-PPEs**	**27**	**37.56**	**1014.00**
	**Aqueous-PPEs**	**36**	**27.83**	**1002.00**
	**Total**	**63**		
**CFU_ECOLI**	**Organic-PPEs**	**27**	**42.63**	**1151.00**
	**Aqueous-PPEs**	**36**	**24.03**	**865.00**
	**Total**	**63**		
**CFU_PAERUGINOSA**	**Organic-PPEs**	**26**	**37.87**	**984.50**
	**Aqueous-PPEs**	**36**	**26.90**	**968.50**
	**Total**	**62**		
**CFU_MIRABILIS**	**Organic-PPEs**	**27**	**19.43**	**524.50**
	**Aqueous-PPEs**	**36**	**41.43**	**1491.50**
	**Total**	**63**		

Test Statistics[a]

	CFU_SAUREUS	CFU_ECOLI	CFU_PAERUGINOSA	CFU_MIRABILIS
**Mann-Whitney U**	**336.000**	**199.000**	**302.500**	**146.500**
**Wilcoxon W**	**1002.000**	**865.000**	**968.500**	**524.500**
**Z**	**-2.252**	**-4.101**	**-2.433**	**-4.771**
**Asymp. Sig. (2-tailed)**	**.024**	**.000**	**.015**	**.000**

a. Grouping Variable: Extract Type

Nonparametric Tests : CFU across solvents

Notes

Output Created		03-NOV-2024 01:31:19
Comments		
Input	Data	C:\Users\CW\Desktop\SPSS_PPE\New folder\PPE2_study.sav
	Active Dataset	DataSet1
	Filter	Concentration < 4 (FILTER)
	Weight	<none>
	Split File	<none>
	N of Rows in Working Data File	63
Syntax		NPTESTS<br>/INDEPENDENT TEST (CFU_SAUREUS CFU_ECOLI CFU_PAERUGINOSA CFU_MIRABILIS) GROUP (Solvent) KRUSKAL_WALLIS(COMPARE=PAIRWISE)<br>/MISSING SCOPE=ANALYSIS USERMISSING=EXCLUDE<br>/CRITERIA ALPHA=0.05 CILEVEL=95.
Resources	Processor Time	00:00:01.06
	Elapsed Time	00:00:01.22

Hypothesis Test Summary

	Null Hypothesis	Test	Sig.	Decision
1	The distribution of CFU_SAUREUS is the same across categories of Solvent.	Independent-Samples Kruskal-Wallis Test	.000	Reject the null hypothesis.
2	The distribution of CFU_ECOLI is the same across categories of Solvent.	Independent-Samples Kruskal-Wallis Test	.000	Reject the null hypothesis.
3	The distribution of CFU_PAERUGINOSA is the same across categories of Solvent.	Independent-Samples Kruskal-Wallis Test	.047	Reject the null hypothesis.
4	The distribution of CFU_MIRABILIS is the same across categories of Solvent.	Independent-Samples Kruskal-Wallis Test	.000	Reject the null hypothesis.

Asymptotic significances are displayed. The significance level is .050.

Independent-Samples Kruskal-Wallis Test

CFU_SAUREUS across Solvent

Independent-Samples Kruskal-Wallis Test Summary

Total N	63
Test Statistic	39.332[a]
Degree Of Freedom	6
Asymptotic Sig.(2-sided test)	.000

a. The test statistic is adjusted for ties.

Pairwise Comparisons of Solvent

Sample 1-Sample 2	Test Statistic	Std. Error	Std. Test Statistic	Sig.	Adj. Sig.[a]
macerate-50%Eth	13.889	7.995	1.737	.082	1.000
MA_PPE-50%Eth	13.889	7.995	1.737	.082	1.000
macerate-100%Eth	38.444	7.995	4.809	.000	.000
MA_PPE-100%Eth	38.444	7.995	4.809	.000	.000
macerate-Acetone	9.333	7.995	1.167	.243	1.000
MA_PPE-Acetone	9.333	7.995	1.167	.243	1.000
macerate-MA_PPE	.000	7.995	.000	1.000	1.000
macerate-infusion	-13.444	7.995	-1.682	.093	1.000
macerate-decoction	-29.889	7.995	-3.738	.000	.004
MA_PPE-infusion	-13.444	7.995	-1.682	.093	1.000
MA_PPE-decoction	-29.889	7.995	-3.738	.000	.004
Acetone-infusion	-4.111	7.995	-.514	.607	1.000
Acetone-50%Eth	4.556	7.995	.570	.569	1.000
Acetone-decoction	-20.556	7.995	-2.571	.010	.213
Acetone-100%Eth	29.111	7.995	3.641	.000	.006
infusion-50%Eth	.444	7.995	.056	.956	1.000
infusion-decoction	16.444	7.995	2.057	.040	.834
infusion-100%Eth	25.000	7.995	3.127	.002	.037
50%Eth-decoction	-16.000	7.995	-2.001	.045	.953
50%Eth-100%Eth	-24.556	7.995	-3.071	.002	.045
decoction-100%Eth	8.556	7.995	1.070	.285	1.000

Each row tests the null hypothesis that the Sample 1 and Sample 2 distributions are the same.
Asymptotic significances (2-sided tests) are displayed. The significance level is .05.
a. Significance values have been adjusted by the Bonferroni correction for multiple tests.

CFU_ECOLI across Solvent

Independent-Samples Kruskal-Wallis Test Summary

Total N	63
Test Statistic	30.867[a]
Degree Of Freedom	6
Asymptotic Sig.(2-sided test)	.000

a. The test statistic is adjusted for ties.

Pairwise Comparisons of Solvent

Sample 1-Sample 2	Test Statistic	Std. Error	Std. Test Statistic	Sig.	Adj. Sig.[a]
macerate-MA_PPE	-2.500	8.398	-.298	.766	1.000
macerate-decoction	-9.611	8.398	-1.144	.252	1.000
macerate-infusion	-10.444	8.398	-1.244	.214	1.000
macerate-50%Eth	12.000	8.398	1.429	.153	1.000
macerate-Acetone	20.611	8.398	2.454	.014	.297
macerate-100%Eth	40.111	8.398	4.776	.000	.000
MA_PPE-decoction	-7.111	8.398	-.847	.397	1.000
MA_PPE-infusion	-7.944	8.398	-.946	.344	1.000
MA_PPE-50%Eth	9.500	8.398	1.131	.258	1.000
MA_PPE-Acetone	18.111	8.398	2.156	.031	.652
MA_PPE-100%Eth	37.611	8.398	4.478	.000	.000
decoction-infusion	-.833	8.398	-.099	.921	1.000
decoction-50%Eth	2.389	8.398	.284	.776	1.000
decoction-Acetone	11.000	8.398	1.310	.190	1.000
decoction-100%Eth	30.500	8.398	3.632	.000	.006
infusion-50%Eth	1.556	8.398	.185	.853	1.000
infusion-Acetone	10.167	8.398	1.211	.226	1.000
infusion-100%Eth	29.667	8.398	3.532	.000	.009
50%Eth-Acetone	-8.611	8.398	-1.025	.305	1.000
50%Eth-100%Eth	-28.111	8.398	-3.347	.001	.017
Acetone-100%Eth	19.500	8.398	2.322	.020	.425

Each row tests the null hypothesis that the Sample 1 and Sample 2 distributions are the same.
Asymptotic significances (2-sided tests) are displayed. The significance level is .05.
a. Significance values have been adjusted by the Bonferroni correction for multiple tests.

CFU_PAERUGINOSA across Solvent

Independent-Samples Kruskal-Wallis Test Summary

Total N	63
Test Statistic	12.758[a]
Degree Of Freedom	6
Asymptotic Sig.(2-sided test)	.047

a. The test statistic is adjusted for ties.

Pairwise Comparisons of Solvent

Sample 1-Sample 2	Test Statistic	Std. Error	Std. Test Statistic	Sig.	Adj. Sig.[a]
MA_PPE-macerate	4.333	8.396	.516	.606	1.000
MA_PPE-infusion	-6.722	8.396	-.801	.423	1.000
MA_PPE-100%Eth	15.944	8.396	1.899	.058	1.000
MA_PPE-decoction	-18.333	8.396	-2.184	.029	.609
MA_PPE-Acetone	19.056	8.396	2.270	.023	.488
MA_PPE-50%Eth	22.722	8.396	2.706	.007	.143
macerate-infusion	-2.389	8.396	-.285	.776	1.000
macerate-100%Eth	11.611	8.396	1.383	.167	1.000
macerate-decoction	-14.000	8.396	-1.668	.095	1.000

macerate-Acetone	14.722	8.396	1.754	.080	1.000
macerate-50%Eth	18.389	8.396	2.190	.029	.599
infusion-100%Eth	9.222	8.396	1.098	.272	1.000
infusion-decoction	11.611	8.396	1.383	.167	1.000
infusion-Acetone	12.333	8.396	1.469	.142	1.000
infusion-50%Eth	16.000	8.396	1.906	.057	1.000
100%Eth-decoction	-2.389	8.396	-.285	.776	1.000
100%Eth-Acetone	-3.111	8.396	-.371	.711	1.000
100%Eth-50%Eth	6.778	8.396	.807	.420	1.000
decoction-Acetone	.722	8.396	.086	.931	1.000
decoction-50%Eth	4.389	8.396	.523	.601	1.000
Acetone-50%Eth	3.667	8.396	.437	.662	1.000

Each row tests the null hypothesis that the Sample 1 and Sample 2 distributions are the same.
Asymptotic significances (2-sided tests) are displayed. The significance level is .05.
a. Significance values have been adjusted by the Bonferroni correction for multiple tests.

CFU_MIRABILIS across Solvent

Independent-Samples Kruskal-Wallis Test Summary

Total N	63
Test Statistic	33.617[a]
Degree Of Freedom	6
Asymptotic Sig.(2-sided test)	.000

a. The test statistic is adjusted for ties.

Pairwise Comparisons of Solvent

Sample 1-Sample 2	Test Statistic	Std. Error	Std. Test Statistic	Sig.	Adj. Sig.[a]
100%Eth-50%Eth	5.778	8.540	.677	.499	1.000
100%Eth-Acetone	-7.167	8.540	-.839	.401	1.000
100%Eth-MA_PPE	-10.278	8.540	-1.204	.229	1.000
100%Eth-decoction	-29.000	8.540	-3.396	.001	.014
100%Eth-infusion	-30.444	8.540	-3.565	.000	.008
100%Eth-macerate	-35.556	8.540	-4.164	.000	.001
50%Eth-Acetone	-1.389	8.540	-.163	.871	1.000
50%Eth-MA_PPE	-4.500	8.540	-.527	.598	1.000
50%Eth-decoction	-23.222	8.540	-2.719	.007	.137
50%Eth-infusion	-24.667	8.540	-2.888	.004	.081
50%Eth-macerate	-29.778	8.540	-3.487	.000	.010
Acetone-MA_PPE	-3.111	8.540	-.364	.716	1.000
Acetone-decoction	-21.833	8.540	-2.557	.011	.222
Acetone-infusion	-23.278	8.540	-2.726	.006	.135
Acetone-macerate	-28.389	8.540	-3.324	.001	.019
MA_PPE-decoction	-18.722	8.540	-2.192	.028	.595
MA_PPE-infusion	-20.167	8.540	-2.362	.018	.382
MA_PPE-macerate	25.278	8.540	2.960	.003	.065
decoction-infusion	-1.444	8.540	-.169	.866	1.000
decoction-macerate	6.556	8.540	.768	.443	1.000
infusion-macerate	5.111	8.540	.599	.549	1.000

Each row tests the null hypothesis that the Sample 1 and Sample 2 distributions are the same.
Asymptotic significances (2-sided tests) are displayed. The significance level is .05.
a. Significance values have been adjusted by the Bonferroni correction for multiple tests.

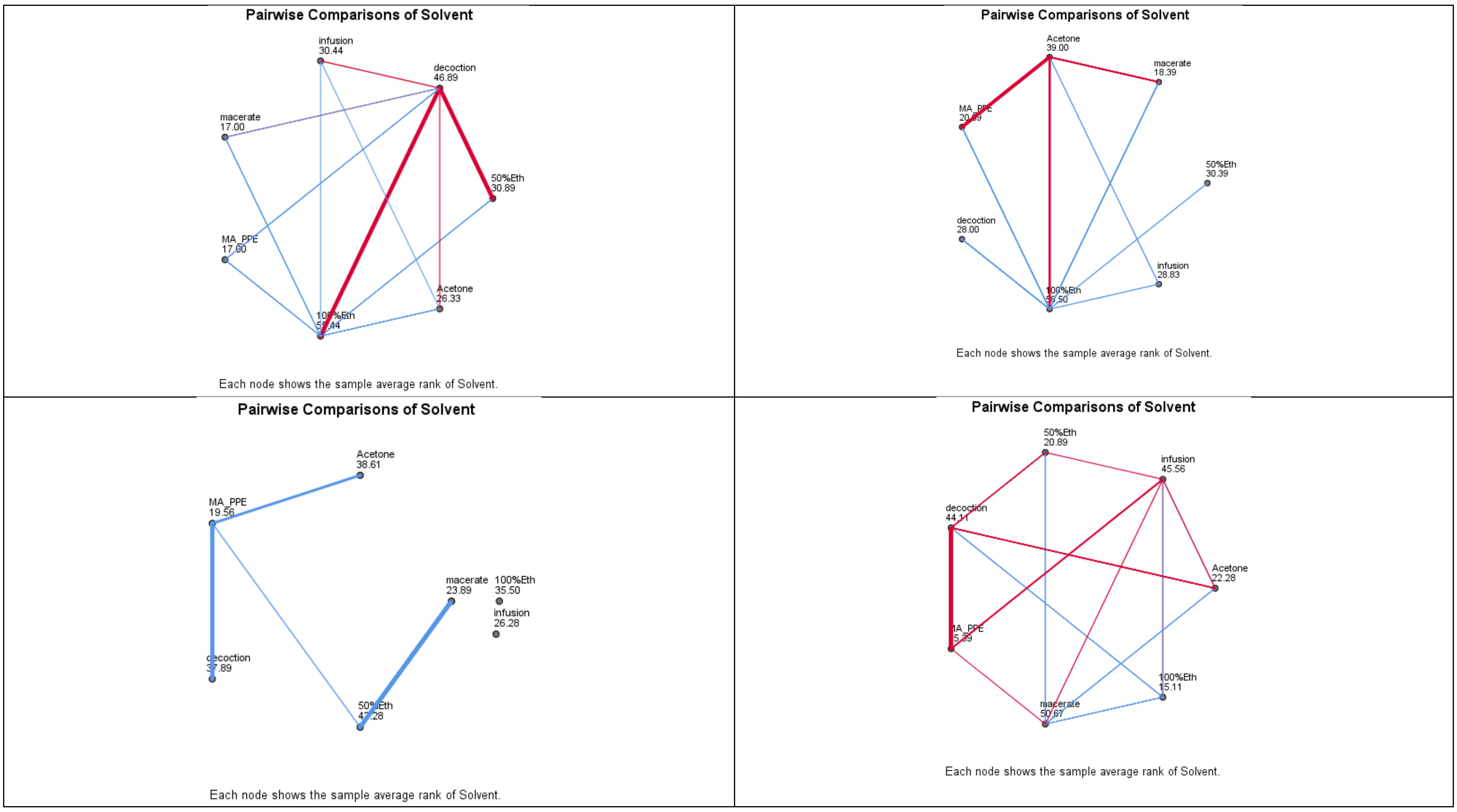

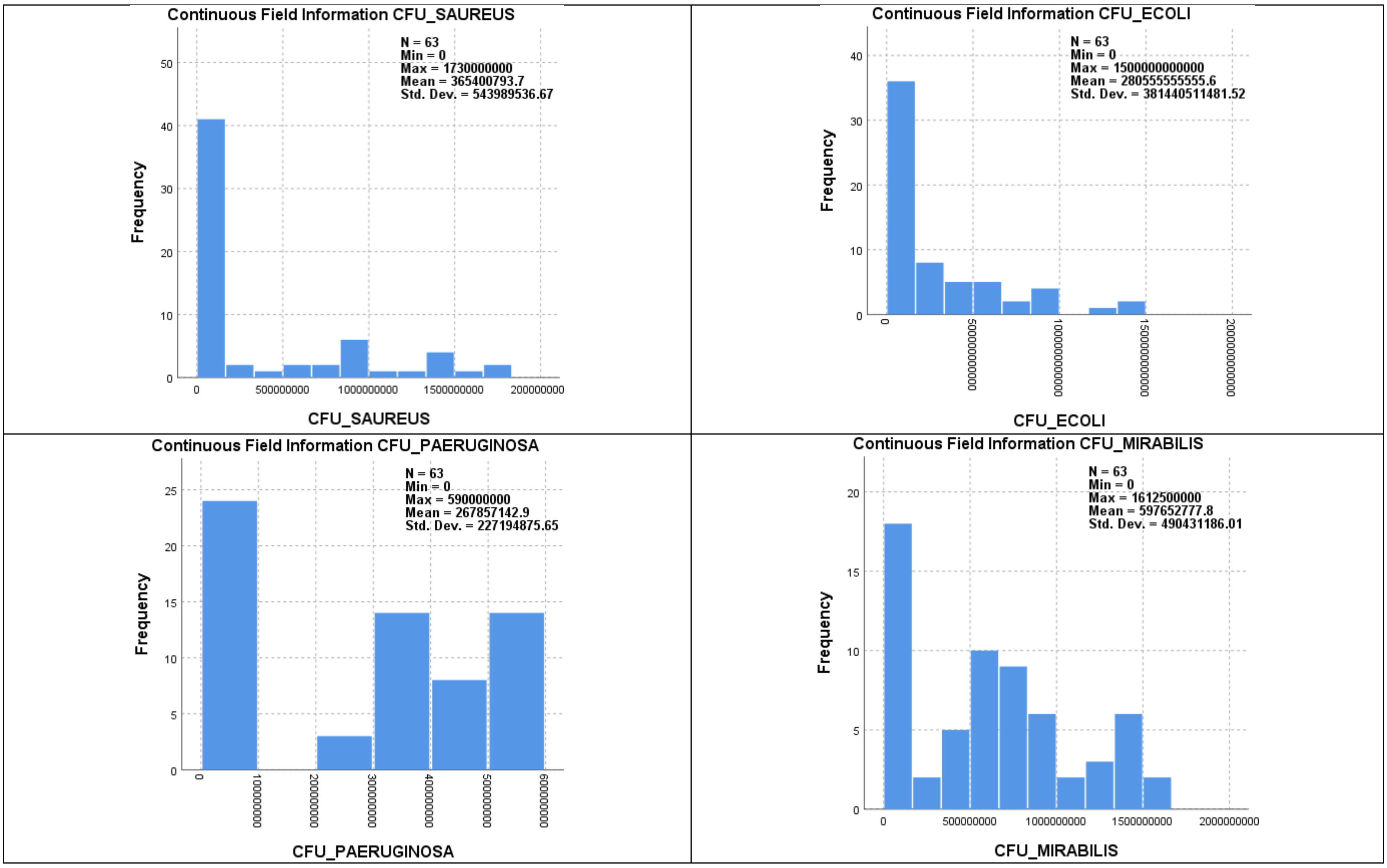

Kruskal-Wallis Test

Ranks

	Solvent	N	Mean Rank
CFU_SAUREUS	50%Eth	9	30.89
	100%Eth	9	55.44
	Acetone	9	26.33
	macerate	9	17.00
	MA_PPE	9	17.00
	decoction	9	46.89
	infusion	9	30.44
	Total	63	
CFU_ECOLI	50%Eth	9	30.39
	100%Eth	9	58.50
	Acetone	9	39.00
	macerate	9	18.39
	MA_PPE	9	20.89
	decoction	9	28.00
	infusion	9	28.83
	Total	63	
CFU_PAERUGINOSA	50%Eth	9	42.28
	100%Eth	9	35.50
	Acetone	9	38.61
	macerate	9	23.89
	MA_PPE	9	19.56
	decoction	9	37.89
	infusion	9	26.28
	Total	63	
CFU_MIRABILIS	50%Eth	9	20.89
	100%Eth	9	15.11
	Acetone	9	22.28
	macerate	9	50.67
	MA_PPE	9	25.39
	decoction	9	44.11
	infusion	9	45.56
	Total	63	

Test Statistics[a,b]

	CFU_SAUREUS	CFU_ECOLI	CFU_PAERUGINOSA	CFU_MIRABILIS
Kruskal-Wallis H	39.332	30.867	12.758	33.617
df	6	6	6	6
Asymp. Sig.	.000	.000	.047	.000

a. Kruskal Wallis Test
b. Grouping Variable: Solvent

CFU/mL in each solvent distributed according to bacterial strain

NPar Tests

Notes

Output Created		10-NOV-2024 09:14:51
Comments		
Input	Data	C:\Users\CW\Desktop\SPSS_PPE\PPE3_study_Multivariate.sav
	Active Dataset	DataSet3
	Filter	<none>
	Weight	<none>
	Split File	<none>
	N of Rows in Working Data File	36
Missing Value Handling	Definition of Missing	User-defined missing values are treated as missing.
	Cases Used	Statistics for each test are based on all cases with valid data for the variable(s) used in that test.
Syntax		NPAR TESTS /K-W=Ethanol_100 Ethanol_50 Acetone Macerate MA_PPE Decoction Infusion BY bacteria(1 4) /MISSING ANALYSIS.
Resources	Processor Time	00:00:00.02
	Elapsed Time	00:00:00.02
	Number of Cases Allowed[a]	241979

a. Based on availability of workspace memory.

[DataSet3] C:\Users\CW\Desktop\SPSS_PPE\PPE3_study_Multivariate.sav

Ranks

	bacteria	N	Mean Rank
Ethanol_100	s.aureus	9	22.89
	e.coli	9	32.00
	p.aeruginosa	9	11.33
	p.mirabilis	9	7.78
	Total	36	
Ethanol_50	s.aureus	9	15.78
	e.coli	9	25.50
	p.aeruginosa	9	17.50
	p.mirabilis	9	15.22
	Total	36	
Acetone	s.aureus	9	13.28
	e.coli	9	25.00
	p.aeruginosa	9	17.28
	p.mirabilis	9	18.44
	Total	36	
Macerate	s.aureus	9	11.00
	e.coli	9	19.00
	p.aeruginosa	9	15.00
	p.mirabilis	9	29.00
	Total	36	
MA_PPE	s.aureus	9	12.50
	e.coli	9	20.00
	p.aeruginosa	9	17.33
	p.mirabilis	9	24.17
	Total	36	
Decoction	s.aureus	9	16.06
	e.coli	9	23.00
	p.aeruginosa	9	11.94
	p.mirabilis	9	23.00
	Total	36	
Infusion	s.aureus	9	10.00
	e.coli	9	24.00
	p.aeruginosa	9	14.00
	p.mirabilis	9	26.00
	Total	36	

Test Statistics[a,b]

	Ethanol_100	Ethanol_50	Acetone	Macerate	MA_PPE	Decoction	Infusion
Kruskal-Wallis H	30.309	6.315	6.207	18.101	8.261	7.258	14.749
df	3	3	3	3	3	3	3
Asymp. Sig.	.000	.097	.102	.000	.041	.064	.002

a. Kruskal Wallis Test

b. Grouping Variable: bacteria

Nonparametric Tests

Hypothesis Test Summary

	Null Hypothesis	Test	Sig.	Decision
1	The distribution of Ethanol_100 is the same across categories of bacteria.	Independent-Samples Kruskal-Wallis Test	.000	Reject the null hypothesis.
2	The distribution of Ethanol_50 is the same across categories of bacteria.	Independent-Samples Kruskal-Wallis Test	.097	Retain the null hypothesis.
3	The distribution of Acetone is the same across categories of bacteria.	Independent-Samples Kruskal-Wallis Test	.102	Retain the null hypothesis.
4	The distribution of Macerate is the same across categories of bacteria.	Independent-Samples Kruskal-Wallis Test	.000	Reject the null hypothesis.
5	The distribution of MA_PPE is the same across categories of bacteria.	Independent-Samples Kruskal-Wallis Test	.041	Reject the null hypothesis.
6	The distribution of Decoction is the same across categories of bacteria.	Independent-Samples Kruskal-Wallis Test	.064	Retain the null hypothesis.
7	The distribution of Infusion is the same across categories of bacteria.	Independent-Samples Kruskal-Wallis Test	.002	Reject the null hypothesis.

Asymptotic significances are displayed. The significance level is .050.

Independent-Samples Kruskal-Wallis Test

Ethanol_100 across bacteria

Independent-Samples Kruskal-Wallis Test Summary

Total N	36
Test Statistic	30.309[a]
Degree Of Freedom	3
Asymptotic Sig.(2-sided test)	.000

a. The test statistic is adjusted for ties.

Pairwise Comparisons of bacteria

Sample 1-Sample 2	Test Statistic	Std. Error	Std. Test Statistic	Sig.	Adj. Sig.[a]
p.mirabilis-p.aeruginosa	3.556	4.927	.722	.470	1.000
p.mirabilis-s.aureus	15.111	4.927	3.067	.002	.013
p.mirabilis-e.coli	24.222	4.927	4.916	.000	.000
p.aeruginosa-s.aureus	11.556	4.927	2.345	.019	.114
p.aeruginosa-e.coli	20.667	4.927	4.195	.000	.000
s.aureus-e.coli	-9.111	4.927	-1.849	.064	.386

Each row tests the null hypothesis that the Sample 1 and Sample 2 distributions are the same.
Asymptotic significances (2-sided tests) are displayed. The significance level is .05.
a. Significance values have been adjusted by the Bonferroni correction for multiple tests.

Ethanol_50 across bacteria
Independent-Samples Kruskal-Wallis Test Summary

Total N	36
Test Statistic	6.315[a,b]
Degree Of Freedom	3
Asymptotic Sig.(2-sided test)	.097

a. The test statistic is adjusted for ties.
b. Multiple comparisons are not performed because the overall test does not show significant differences across samples.

Acetone across bacteria
Independent-Samples Kruskal-Wallis Test Summary

Total N	36
Test Statistic	6.207[a,b]
Degree Of Freedom	3
Asymptotic Sig.(2-sided test)	.102

a. The test statistic is adjusted for ties.
b. Multiple comparisons are not performed because the overall test does not show significant differences across samples.

Macerate across bacteria

Independent-Samples Kruskal-Wallis Test Summary

Total N	36
Test Statistic	18.101[a]
Degree Of Freedom	3
Asymptotic Sig.(2-sided test)	.000

a. The test statistic is adjusted for ties.

Pairwise Comparisons of bacteria

Sample 1-Sample 2	Test Statistic	Std. Error	Std. Test Statistic	Sig.	Adj. Sig.[a]
s.aureus-p.aeruginosa	-4.000	4.447	-.899	.368	1.000
s.aureus-e.coli	-8.000	4.447	-1.799	.072	.432
s.aureus-p.mirabilis	-18.000	4.447	-4.047	.000	.000
p.aeruginosa-e.coli	4.000	4.447	.899	.368	1.000
p.aeruginosa-p.mirabilis	-14.000	4.447	-3.148	.002	.010
e.coli-p.mirabilis	-10.000	4.447	-2.249	.025	.147

Each row tests the null hypothesis that the Sample 1 and Sample 2 distributions are the same.
Asymptotic significances (2-sided tests) are displayed. The significance level is .05.
a. Significance values have been adjusted by the Bonferroni correction for multiple tests.

MA_PPE across bacteria

Independent-Samples Kruskal-Wallis Test Summary

Total N	36
Test Statistic	8.261[a]
Degree Of Freedom	3
Asymptotic Sig.(2-sided test)	.041

a. The test statistic is adjusted for ties.

Pairwise Comparisons of bacteria

Sample 1-Sample 2	Test Statistic	Std. Error	Std. Test Statistic	Sig.	Adj. Sig.[a]
s.aureus-p.aeruginosa	-4.833	4.167	-1.160	.246	1.000
s.aureus-e.coli	-7.500	4.167	-1.800	.072	.431
s.aureus-p.mirabilis	-11.667	4.167	-2.800	.005	.031
p.aeruginosa-e.coli	2.667	4.167	.640	.522	1.000
p.aeruginosa-p.mirabilis	-6.833	4.167	-1.640	.101	.606
e.coli-p.mirabilis	-4.167	4.167	-1.000	.317	1.000

Each row tests the null hypothesis that the Sample 1 and Sample 2 distributions are the same.
Asymptotic significances (2-sided tests) are displayed. The significance level is .05.
a. Significance values have been adjusted by the Bonferroni correction for multiple tests.

Decoction across bacteria

Independent-Samples Kruskal-Wallis Test Summary

Total N	36
Test Statistic	7.258[a,b]
Degree Of Freedom	3
Asymptotic Sig.(2-sided test)	.064

a. The test statistic is adjusted for ties.
b. Multiple comparisons are not performed because the overall test does not show significant differences across samples.

Infusion across bacteria

Independent-Samples Kruskal-Wallis Test Summary

Total N	36
Test Statistic	14.749[a]
Degree Of Freedom	3
Asymptotic Sig.(2-sided test)	.002

a. The test statistic is adjusted for ties.

Pairwise Comparisons of bacteria

Sample 1-Sample 2	Test Statistic	Std. Error	Std. Test Statistic	Sig.	Adj. Sig.[a]
s.aureus-p.aeruginosa	-4.000	4.927	-.812	.417	1.000
s.aureus-e.coli	-14.000	4.927	-2.842	.004	.027
s.aureus-p.mirabilis	-16.000	4.927	-3.248	.001	.007
p.aeruginosa-e.coli	10.000	4.927	2.030	.042	.254
p.aeruginosa-p.mirabilis	-12.000	4.927	-2.436	.015	.089
e.coli-p.mirabilis	-2.000	4.927	-.406	.685	1.000

Each row tests the null hypothesis that the Sample 1 and Sample 2 distributions are the same.
Asymptotic significances (2-sided tests) are displayed. The significance level is .05.
a. Significance values have been adjusted by the Bonferroni correction for multiple tests.

CONCLUSION

1. Difference between aqueous-PPEs and organic-PPEs

The results indicated that CFU/mL had significant difference between Organic-PPEs and Aqueous-PPEs, where its levels were significantly reduced in aqueous -PPEs when considering *s. aureus* [z=-2.252, p= .024], *e.coli* [z= -4.101, p < .001], and *p. aerugenosa* [z= -2.43, p= .015], while they were significantly reduced in the organic-PPEs when considering *p.mirabilis* [z= -4.771, p< .001].

2. Difference between solvent

A Kruskal-Wallis test indicated that there was a significant difference in [CFU/mL in s.aureus across seven solvent types, χ^2 (6, 63) = 39.33 *p* = < .001. Post-hoc comparisons using Dunn's method with a Bonferroni correction for multiple tests indicated that the mean rank of s.aureus' CFU/mL in absolute ethanol (55.44) was significantly higher than that of the aqueous PPEs solvents including infusion, macerate and MA-PPE, as well as being significantly higher than the other two solvents within the organic-PPEs with *p values range* = [<.001- 0.045]

Based on Kruskal-Wallis test, Post-hoc comparisons using Dunn's method with a Bonferroni correction for multiple tests indicated that:

- the mean rank of *s.aureus'* CFU/mL in absolute ethanol (55.44) was significantly higher than that of the macerate (p <0.001), MA_PPE p <0.001, infusion (p=0.04)
- S. aureus: 100% Ethanol shows significant higher mean rank than 50% ethanol (p = 0.045) and Acetone (p=0.006) from the Organic-PPEs
- S.aureus: macerate and MA-PPE had equivalent mean ranks of 17 and both were significantly different from: absolute ethanol, and decoction extracts.
- E. coli: 100% Ethanol shows significant higher mean rank (55.50) than all the aqueous PPEs (i,e,macerate, MA_PPE, decoction, and infusion [p= <0.001, < 0.001, 0.006, 0.009 respectively]) as well as a difference to 50% ethanolic extract from the Organic-PPEs (p=0.017). In this bacterial strain aqueous-PPEs are more effective
- P. aeruginosa: No significant differences
- P. mirabilis: Strong differences between organic-PPEs and aqueous PPEs, where the organic-PPEs are more effective than the aqueous PPEs. But here the aqueous PPEs have higher mean ranks of CFU/mL and there is a significant difference between
    - 100%Eth-decoction p=.014
    - 100%Eth-infusion p=.008
    - 100%Eth-macerate p= .001
    - 50%Eth-macerate p=0.010
    - and Acetone-macerate p= .019.
    - still the MA-PPE is not significantly different from the active organic-PPEs.

Figures:

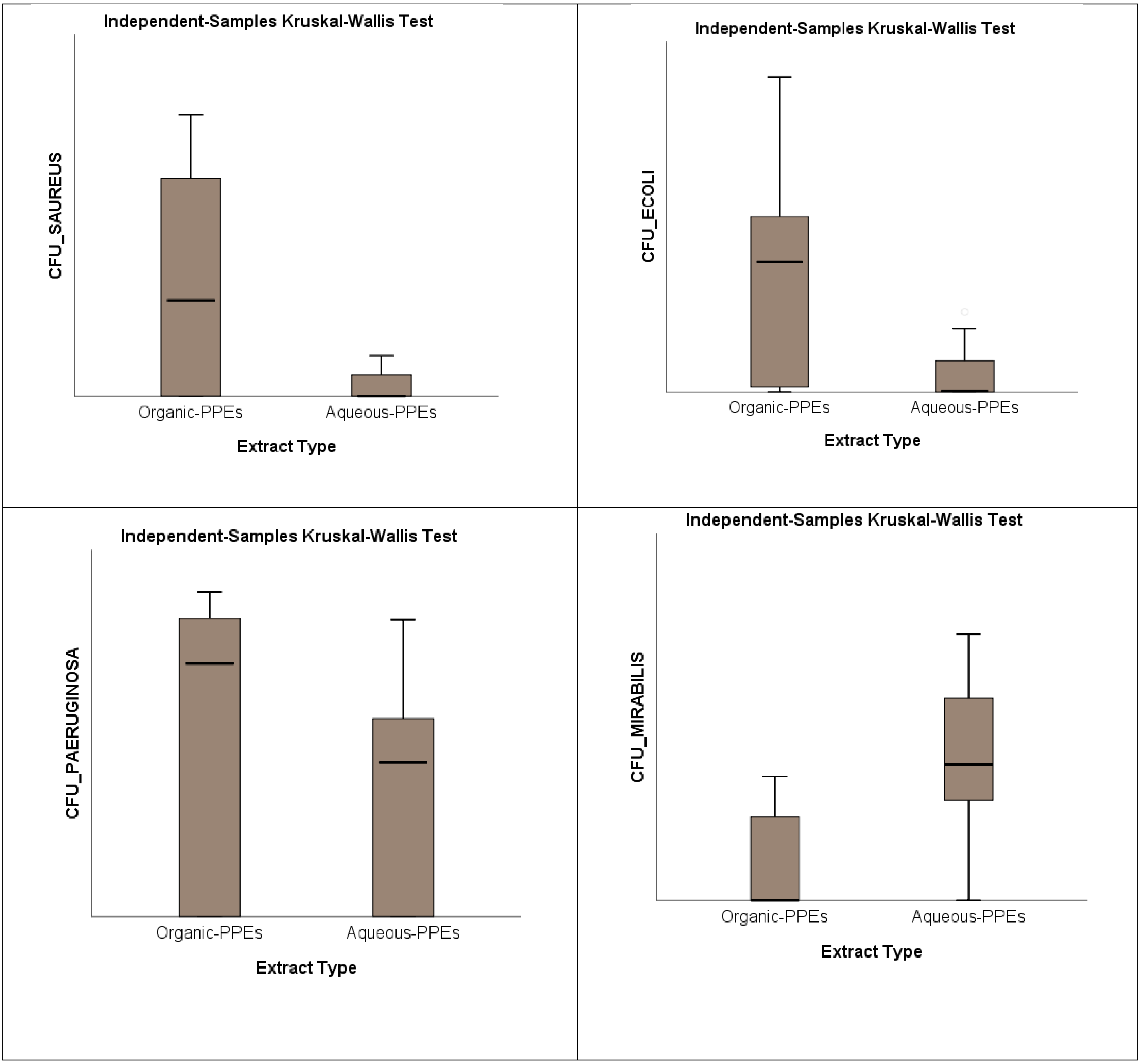
Independent-Samples Kruskal-Wallis Test
CFU_SAUREUS
Organic-PPEs
Aqueous-PPEs
Extract Type
Independent-Samples Kruskal-Wallis Test
CFU_ECOLI
Organic-PPEs
Aqueous-PPEs
Extract Type
Independent-Samples Kruskal-Wallis Test
CFU_PAERUGINOSA
Organic-PPEs
Aqueous-PPEs
Extract Type
Independent-Samples Kruskal-Wallis Test
CFU_MIRABILIS
Organic-PPEs
Aqueous-PPEs
Extract Type

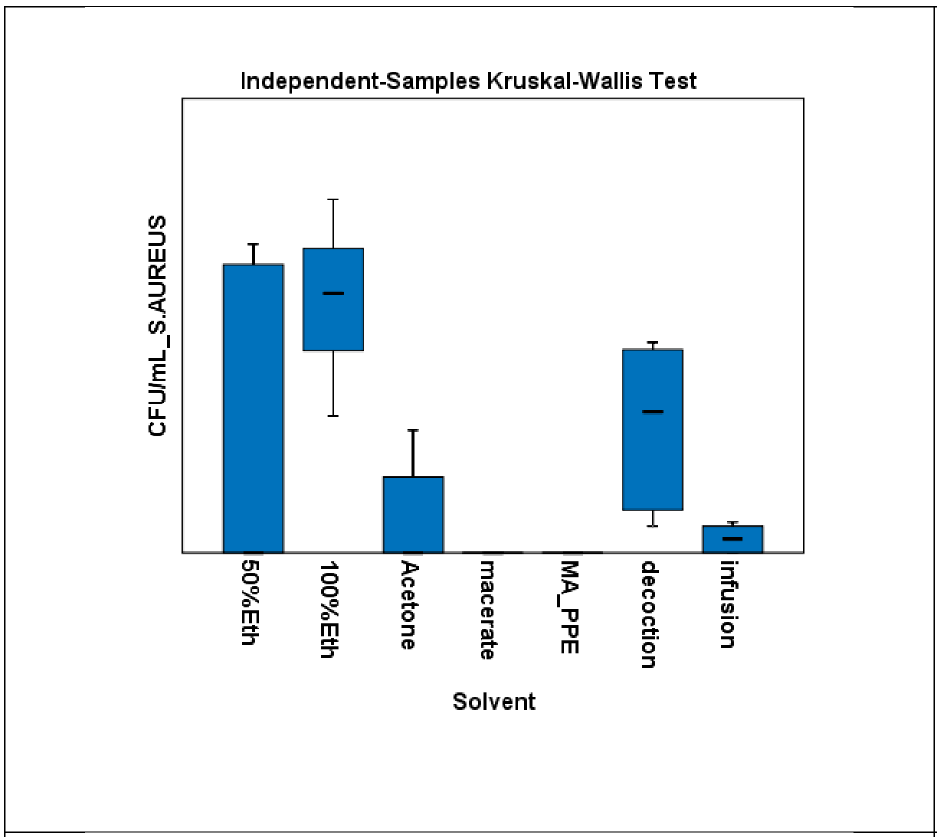
Independent-Samples Kruskal-Wallis Test
CFU/mL_S.AUREUS
50%Eth
100%Eth
Acetone
macerate
MA_PPE
decoction
infusion
Solvent

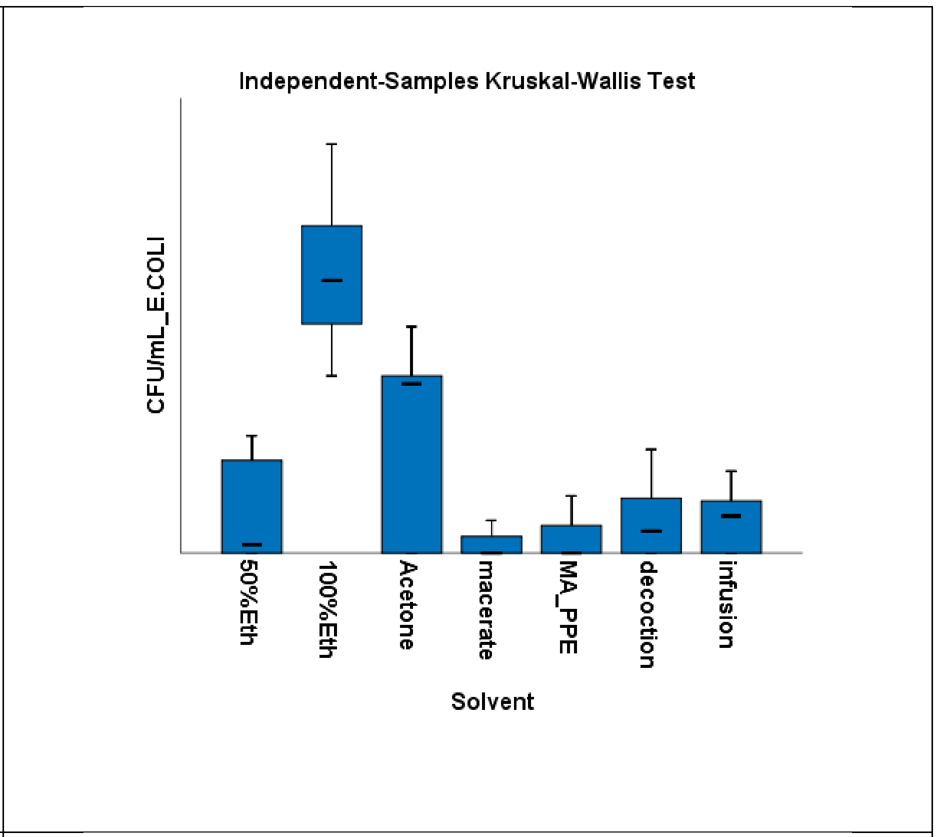
Independent-Samples Kruskal-Wallis Test
CFU/mL_E.COLI
50%Eth
100%Eth
Acetone
macerate
MA_PPE
decoction
infusion
Solvent

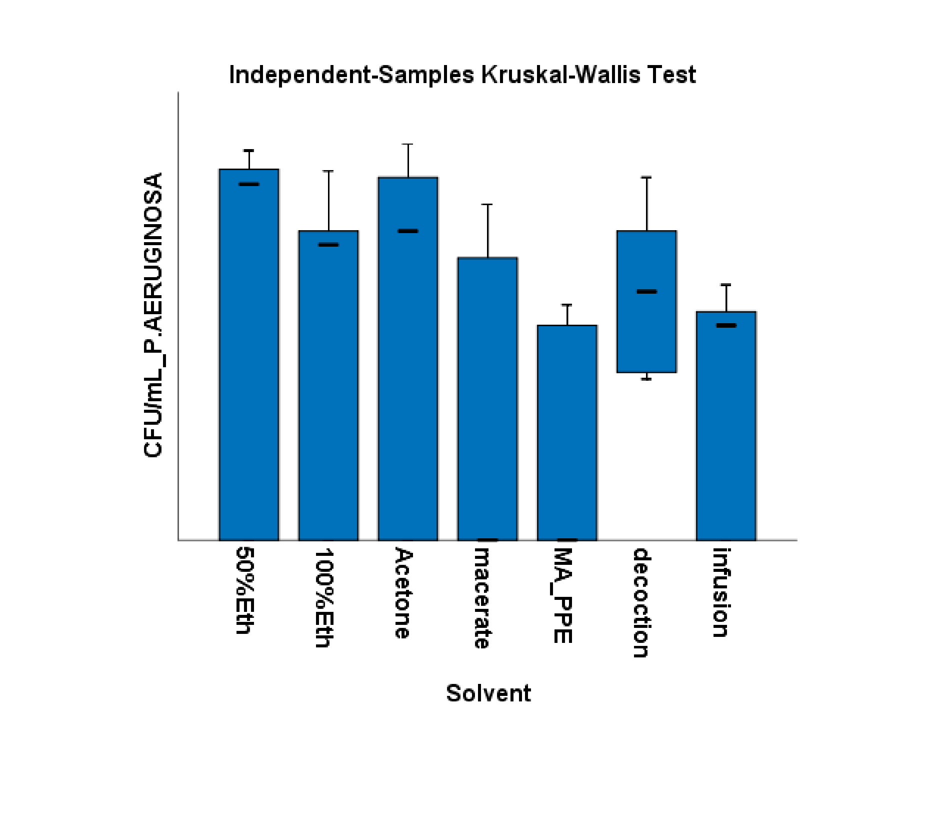
Independent-Samples Kruskal-Wallis Test
CFU/mL_P.AERUGINOSA
50%Eth
100%Eth
Acetone
macerate
MA_PPE
decoction
infusion
Solvent

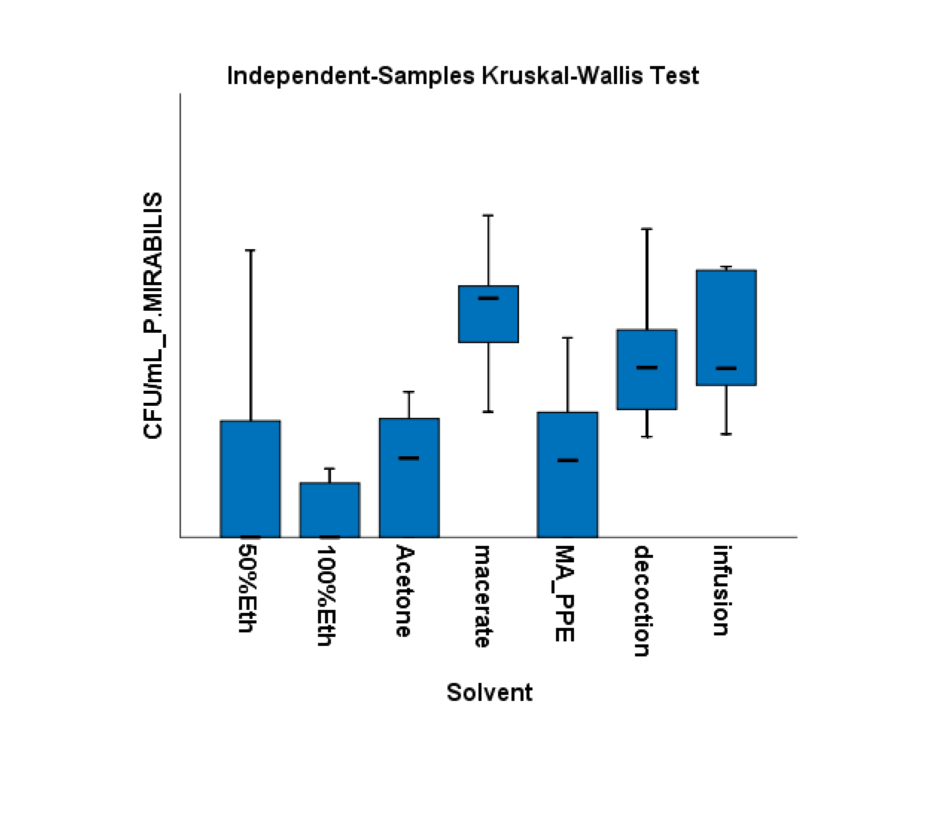
Independent-Samples Kruskal-Wallis Test
CFU/mL_P.MIRABILIS
50%Eth
100%Eth
Acetone
macerate
MA_PPE
decoction
infusion
Solvent
